# Tailoring of Colloidal HfO_2_ Nanocrystals with Unique Morphologies and New Self‐Assembly Features

**DOI:** 10.1002/smsc.202300209

**Published:** 2024-01-24

**Authors:** Thorsten Ohlerth, Hongchu Du, Thomas Hammoor, Joachim Mayer, Ulrich Simon

**Affiliations:** ^1^ Institute for Inorganic Chemistry, RWTH Aachen University and JARA ‐ Fundamentals of Future Information Technologies Aachen 52074 Germany; ^2^ Ernst Ruska‐Centre for Microscopy and Spectroscopy with Electrons, Forschungszentrum Jülich GmbH, Central Facility for Electron Microscopy RWTH Aachen University and JARA ‐ Fundamentals of Future Information Technologies 52425 Jülich Germany

**Keywords:** growth control, ligands, liquid crystals, nanoparticles, oxides, self‐assembly

## Abstract

Advancing the synthesis of HfO_2_ nanocrystals, a refined anhydrous protocol that enables kinetic trapping of the metastable tetragonal phase and modulates twinning defects in anisotropically grown monoclinic nanoprisms is presented. This evolved sol–gel approach examines the role of the capping agent tri‐*n*‐octylphosphine oxide (TOPO) and introduces a novel heating strategy with sequential growth stages. Replacement of TOPO with triphenylphosphine oxide (TPPO) leads to the formation of prismatic hafnia nanocrystals exhibiting pristine {011}‐facets of the monoclinic phase. Furthermore, a hot‐injection inspired heating approach yields sub‐4 nm isotropic HfO_2_ nanocrystals in the tetragonal phase, bypassing the need for aliovalent cation species. In contrast, a heat‐up approach culminates in the generation of well‐characterized HfO_2_ nanorods. With sophisticated transmission electron microscopy analysis and the Wulff construction method, insights into the structural nucleation of nanoparticle growth are provided. This synthesis offers exceptional control and facilitates the formation of self‐assemblies akin to liquid crystals, opening the door for new applications with nanocolloidal HfO_2_.

## Graphical abstract

A refined sol–gel method for HfO_2_ nanocrystals synthesis traps the tetragonal phase in a hot‐injection heating protocol and studies the role of capping agents like tri‐*n*‐octylphosphine oxide (TOPO) and triphenylphosphine oxide (TPPO). The heat‐up heating strategy allows for better morphology control and a consecutive growth approach. Advanced transmission electron microscopy analysis elucidates nanoparticle nucleation. The nanocrystals form self‐assemblies, suggesting innovative applications for colloidal HfO_2_.

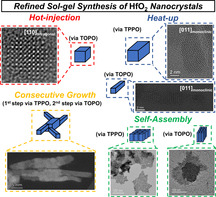

## 1 Introduction

The development of new nanomaterials that exhibit size dependent characteristics, opens new opportunities of application in fields like medicine, electronics, catalysis, optics, or energy storage.^[^ ^1^, ^2^, ^3^, ^4^, ^5^ ^]^ One of the most remarkable features of nanomaterials, including pure metals as well as multinary compounds, relies on quantum size effects and the tendency to transform into metastable crystal phases at ambient conditions, which can usually only be observed e.g., at elevated temperatures, high pressures or through doping.^[^ ^6^, ^7^ ^]^ The origin for this crystallographic change in phase stability is based on the ratio of surface‐to‐lattice atoms, which is negligibly small in the bulk phase, but considerably larger for nanomaterials. By that, the thermodynamic properties of a material are increasingly governed by surface and interfacial free energy terms and less by the bulk free energy. In case of anisotropically shaped nanoparticles, especially for so called one‐dimensional (1D) or two‐dimensional (2D) materials, the surface to volume ratio and twinning phenomena can have an even stronger impact on the characteristics of the material.^[^ ^8^, ^9^ ^]^

Hafnia, as a high‐*κ*‐dielectric material, has risen awareness when Müller et al. found that it exhibits ferroelectric behavior in thin films with sizes of about 10 nm thickness.^[^ ^10^ ^]^ They explained this phenomenon to occur based on a metastable orthorhombic phase (space group *Pbc*2_1_) that is polarized due to oxygen‐site changes in the crystal system. In fact, HfO_2_ displays even more crystal phases: the thermodynamically favored monoclinic (M) phase (space group *P* 2_1_/c) crystallizes under ambient conditions in bulk materials and transforms to a tetragonal (T) phase (space group *P* 4_2_/*nmc*) at ≈1700 °C and eventually to the cubic fluorite type phase at ≈2600 °C (space group $Fm\bar{3}m$).^[^ ^11^, ^12^ ^]^ As mentioned above, the two latter phases can be stabilized when hafnia is confined to nanoscale domains. Hunter et al. predicted that the T phase of HfO_2_ is thermodynamically favored at a critical crystallite size below ≈3.6 nm.^[^ ^13^ ^]^

Over the last two decades, HfO_2_ has already been explored as a high‐*κ* dielectric and a further extension of its application in high performance electronics is conceivable, if the metastable phases of hafnia could be obtained at ambient conditions, as e.g., the dielectric permittivity value of 16 for the monoclinic phase rises to either 29 or 70 for the tetragonal and cubic system, respectively.^[^ ^14^ ^]^ Meanwhile, HfO_2_ has also attracted high attention for nonvolatile high‐density memory technologies as well as for neuromorphic devices, which is comprehensively been summarized in a very recent review article by Banerjee et al.^[^ ^15^ ^]^ Beyond this, Wang et al. as well as our group have explored the application of self‐assembling HfO_2_ nanocrystals for volatile as well as non‐volatile resistive switching, which may further expand the technological impact of HfO_2_.^[^ ^16^, ^17^ ^]^

By now, several methods have been applied to chemically synthesize monoclinic or cubic HfO_2_ nanocrystals with different sizes.^[^ ^18^, ^19^, ^20^, ^21^, ^22^, ^23^, ^24^, ^25^, ^26^, ^27^, ^28^, ^29^, ^30^ ^]^ Typical high‐boiling solvents in non‐aqueous syntheses are benzyl alcohol, benzylamine, and oleylamine. Apart from these approaches, presumably one of them stands out as it features a non‐hydrolytic gram scale synthesis of sub‐4 nm nanocrystalline hafnia, where tri‐*n*‐octylphoshphine oxide (TOPO) serves as both, a high boiling solvent for the metal precursors and as coordinating agent that prevents crystal aggregation.^[^ ^31^ ^]^ The metal precursors consist of an equimolar amount of metal halides and metal alkoxides that undergo a condensation‐like reaction, which was first introduced by Colvin in TOPO.^[^ ^32^, ^33^, ^34^ ^]^ This approach is not only limited to produce HfO_2_, but also to synthesize ZrO_2_ and Hf_*x*_Zr_1*−x*_O_2_ mixtures via a cross‐condensation mechanism between both metals.^[^ ^31^, ^35^ ^]^ Pure HfO_2_ nanocrystals were obtained in two variants: One particle class yielded spherical crystallites with a diameter of approximately 4 nm in the T phase when heated to 360 °C, the other delivered a rod‐like morphology as M phase nanorods (HfO_2_‐NR) when the temperature was even further increased toward 400 °C. Yet, reproducing T phase nanocrystals was somewhat contradictory in follow‐up researches involving this specific sol–gel method, and stabilization of the T phase involved reactions with aliovalent metal species.^[^ ^36^, ^37^, ^38^, ^39^, ^40^ ^]^ The proneness of hafnia to undergo a phase transformation from T to M phase is believed to result from the greater difference of these two phases in bulk free energies Δ*G*_bulk_ compared to zirconia. While Δ*G*_bulk_ for HfO_2_ is 194 meV and just 140 meV for ZrO_2_, the difference in surface free energies (*γ*) for both systems can be neglected and by that the critical size for the stabilization of the T phase in hafnia is lower than for zirconia.^[^ ^41^ ^]^

It was suggested that a martensitic growth mechanism explains the transformation from T crystals to M anisotropic crystals within hot liquid solution.^[^ ^31^, ^35^ ^]^ While maintaining the approximate shape of the initial nucleus, the atoms shift diffusionless in order to establish the less symmetrical M crystal phase, inducing a collateral shear strain in the system. The martensitic transformation from the tetragonal to the monoclinic phase is associated with a change of the metal coordination number from 8 to 7 and for half of oxygen atoms it changes from 4 to 3. In case of HfO_2_, the crystal system counters the shear strain by generating one or multiple twinning layers alongside the *<*100*>*_M_ growing direction of the anisotropic HfO_2_‐NR. In contrast, we have recently shown that shear strain can be avoided through a layer‐by‐layer growth, in which a surface T‐like phase was formed under kinetic conditions during solvothermal growth of M nanocrystals.^[^ ^42^ ^]^ A surface T‐to‐M phase transition at the unit cell level requires marginal shearing. The phase transition may result in the formation of twin boundaries. They are coherent interfaces across which the two twin parts of the monoclinic phase show no breaking their neighbor atom connectivity.^[^ ^42^ ^]^ With disregard of shearing, twinning itself will lead to an increase of the system energy. First, it results in a formation of the ferroelectric *Pbc*2_1_ phase at the twin boundary through a two‐fold screw twin operation. This phase has a higher total energy than the M phase. Second, the *Pbc*2_1_ structure is strained because its lattice parameters are different from those of the M phase. Interestingly, the energy and lattice strain caused by individual twinning can be reduced by unit‐cell‐wise consecutive twinning. The latter leads to a formation of an anti‐polar orthorhombic (space group *Pbca*) phase, whose total energy and lattice parameters are very close to that of the M phase.^[^ ^42^ ^]^ However, in these studies the role of the stabilizing ligands on the phase, size, and shape formation are not systemically explored.

In TOPO, HfO_2_ nanoparticles grow along the *<*100*>* direction but typically high surface energy {011} facets are not well‐developed. This contrasts hafnia films, which are formed via atomic layer deposition and which show the expected $\left\{\bar{1}11\right\}$‐ and {111} facets, as they are low in their *γ* values.^[^ ^43^ ^]^ It would therefore be interesting to explore, how far the crystal formation of colloidal hafnia can further be controlled by subtle changes in the ligand polarity. We therefore introduced triphenylphosphine oxide (TPPO) as a ligand bearing different polarity but the same P=O headgroups to coordinate to the hafnia surface as TOPO. We thereby expect that the addition of TPPO may impact the gelation, monomer formation, mass transport, and surface adherence. In order to systematically interrogate the formation process, we designed the following experimental approach: 1) comparing the effect of TOPO and TPPO on nanocrystal formation and surface shaping, 2) comparing a hot‐injection and a heat‐up protocol, 3) applying a consecutive synthesis protocol which is based on (1) and (2) in order to explore effects of heterogeneous crystal formation. Our studies revealed that rational design of the crystal morphology and size of the nanocrystals is achievable to a large extent and we observed intriguing self‐assembling topologies that are yet unprecedented for colloidal hafnia.

## 2 Results

### 2.1 Hot‐Injection and Heat‐Up in TOPO and TPPO

**Figure 1:**
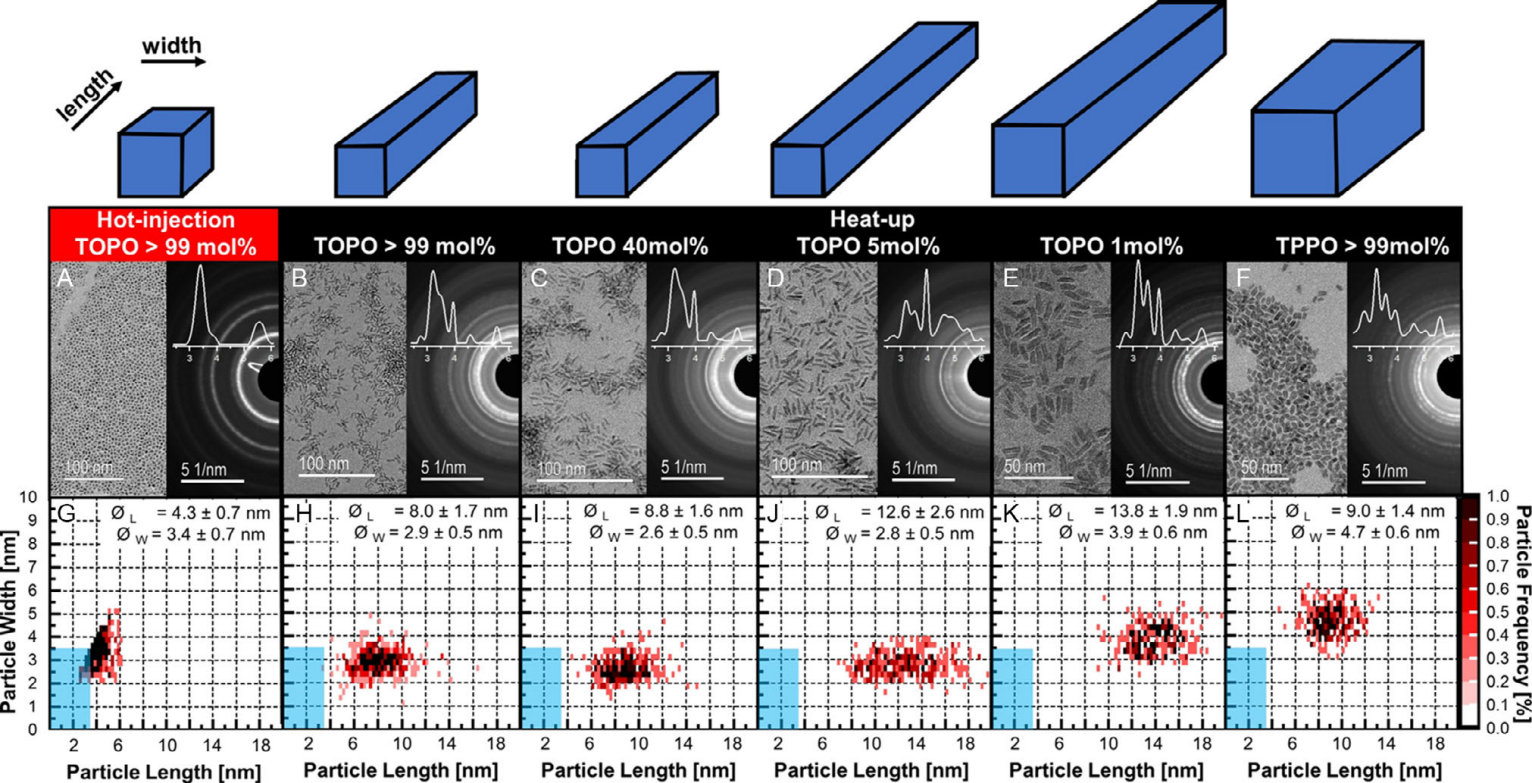
Upper panel: Bright‐field TEM images and selected area electron diffractograms (SAEDs) of the synthesized nanocrystals. A) HI‐TOPO, B) HU‐TOPO, C) HU‐40TOPO, D) HU‐5TOPO, E) HU‐1TOPO, and F) HU‐TPPO. Radially averaged intensity profiles were superposed in the SAEDs. Lower panel:(G‐L) show correspondingly the particle size frequency analysis, where cyan fields highlight the predicted critical threshold of 3.6 nm for the tetragonal (T) phase.^[^ ^13^ ^]^ The initials “HI” and “HU” denote, respectively, the hot‐injection and heat‐up strategies. The blue cuboids depict the approximated respective particle morphology.

The characteristics of HfO_2_ nanoparticles synthesized using different reaction protocols are summarized in **Table** **1**. **Figure** **1** shows conventional transmission electron microscopy (TEM) images, size distribution heatmaps, and selected area electron diffractograms (SAEDs) for nanoparticles that were synthesized using hot‐injection and heat‐up strategies with varied amounts of TOPO and TPPO. It should be noted that in order to generate a complete mixing of the compounds, the molar amounts of TOPO and TPPO were adjusted, resulting in different equivalents of phosphine oxide to hafnium. The size distribution statistics were calculated from measurements of more than 300 individual nanoparticles in TEM images at a 100 k or higher magnification.

**Table 1:** Summary of the synthesized HfO_2_ nanoparticles

Sample denotationa)	Synthesis strategy	TOPO	TPPO	Length	Width	Length	Crystal	Remarks
		[mmol]	[mmol]	[nm]	[nm]	Width	phase	
HI‐TOPO	Hot‐injection at 340 °C	26.0	–	4.3 ± 0.7	3.4 ± 0.7	1.3 ± 0.5	tetragonal	dots, faceted
HI‐TOPO (390 °C)	Hot‐injection at 390 °C	26.0	–	3.7 ± 0.7	2.9 ± 0.5	1.3 ± 0.5	tetragonal	dots, faceted
HU‐TOPO	Heat‐up to 340 °C	26.0	–	8.0 ± 1.7	2.9 ± 0.5	2.8 ± 1.1	monoclinic	rods, twinning
HU‐TOPO (2^nd^)	2^nd^ precursor addition then Heat‐up to 340 °C	26.0	–	14.5 ± 5.7	2.9 ± 0.5	5.0 ± 2.8	monoclinic	rods, twinning
HU‐TOPO (3^rd^)	3^rd^ precursor addition then Heat‐up to 340 °C	26.0	–	21.6 ± 7.0	2.9 ± 0.5	7.4 ± 3.7	monoclinic	rods, twinning
HU‐40TOPO	Heat‐up to 340 °C	13.0	19.5	8.8 ± 1.6	2.6 ± 0.5	3.4 ± 1.3	monoclinic	rods, twinning
HU‐5TOPO	Heat‐up to 340 °C	2.0	39.0	12.6 ± 2.6	2.8 ± 0.5	4.5 ± 1.7	monoclinic	rods, twinning
HU‐1TOPO	Heat‐up to 340 °C	0.5	39.0	13.8 ± 1.9	3.9 ± 0.6	3.5 ± 1.0	monoclinic	rodlike, prismatic, and occasional twinning
HU‐TPPO	Heat‐up to 340 °C	–	39.0	9.0 ± 1.4	4.7 ± 0.6	1.9 ± 0.5	monoclinic	prisms, twinning at NC end
HU‐TPPO (2^nd^)	2^nd^ precursor addition then Heat‐up to 340 °C	–	39.0	11.2 ± 1.7	5.9 ± 0.9	1.9 ± 0.6	monoclinic	prisms, twinning at NC end
HU‐TPPO(3^rd^)	3^rd^ precursor addition then Heat‐up to 340 °C	–	39.0	12.5 ± 2.6	6.6 ± 0.8	1.9 ± 0.6	monoclinic	prisms, twinning at NC end
HU‐TPPO⟲TOPO	Ligand exchange (see main text)	5.2	39.0	13.0 ± 4.2	6.6 ± 1.4	2.0 ± 1.1	monoclinic	rods, prisms, and “nanohybrides” (HfO_2_‐NH)

a) The prefix “HI” and “HU” stand for hot‐injection and heat‐up protocols, respectively. The number leading “TOPO” indicates its portion (mol%) in the TOPO and TPPO surfactant mixtures, while “TPPO⟲TOPO” shall emphasize the ligand exchange from TPPO to TOPO.

TEM imaging revealed that nanocrystals synthesized using the hot‐injection in TOPO are nanodots with length‐width aspect ratios of about 1.3, whereas all the nanocrystals synthesized using the heat‐up method are elongated particles, i.e., nanorods and nanoprisms, with aspect ratios of about 2 and above, irrespective of the surfactants and their amount. The SAED (Figure 1A) and X‐ray diffraction data (see Figure S1, Supporting Information) suggest that the nanodots appear to be stabilized in the high temperature tetragonal phase of HfO_2_.^[^ ^11^, ^12^ ^]^ In contrast, all other synthesized nanorods and nanoprisms can be assigned to the room temperature stable monoclinic phase (Figure 1B–F). A more detailed discussion of the x‐ray and electron diffraction results is found in Figure S2 and S3, Supporting Information.

For reactions conducted in pure TOPO, the length‐width aspect ratio of the nanorods synthesized using the heat‐up method (Figure 1B,H) is about 2.8, compared to an aspect ratio of 1.3 for the slightly elliptic nanodots obtained from the hot‐injection method (Figure 1A,G). In contrast, for TPPO no essential differences were observed between the hot‐injection and heat‐up methods in terms of the size and morphology. The nanoprisms (HfO_2_‐NP) have well‐defined facets that are parallel to their long axis.

They are therefore distinguished from the nanorods throughout this report, where the latter are elongated nanocrystals without this pristine faceting behavior (albeit their cross section is not necessarily a round shape). The determination of the shape and facets of the nanoprisms shall be described later. The aspect ratio of HfO_2_‐NP grown in TPPO is close to 2 (Table 1 and Figure 1E,F), being smaller than 2.8 of the HfO_2_‐NR obtained from TOPO by the same heat‐up method.

For heat‐up reactions in a mixture of TOPO and TPPO, the length and width of the grown nanorods were gradually increased from 9 and 3 nm to 14 and 4 nm, respectively, with a decrease of the fraction of TOPO in the mixture from 40 to 1 mol% (Table 1 and Figure 1B–E). The trend of increasing width of the nanocrystals was maintained, while the increase in length was reversed when the fraction of TOPO was reduced to zero, i.e., in the case of pure TPPO. The size and shape and their distribution for HfO_2_‐NR grown in a mixture with 40 mol% TOPO are comparably the same as those of the pure TOPO case. In contrast, nanorods grown in 5 mol% of TOPO are about 13 nm in length, being considerably longer than that of about 9 nm for nanorods obtained from 40 mol% of TOPO. Further reducing the TOPO to 1 mol% resulted in a further increase in length (≈14 nm) and width (≈4 nm). But without TOPO, i.e., in the extreme case of pure TPPO, the length (≈9 nm) of the synthesized nanoprisms becomes ≈50% shorter while their width (≈5 nm) ≈20% broader compared to the case of mixture with only 1 mol% of TOPO.

### 2.2 Consecutive Growth

**Figure 2:**
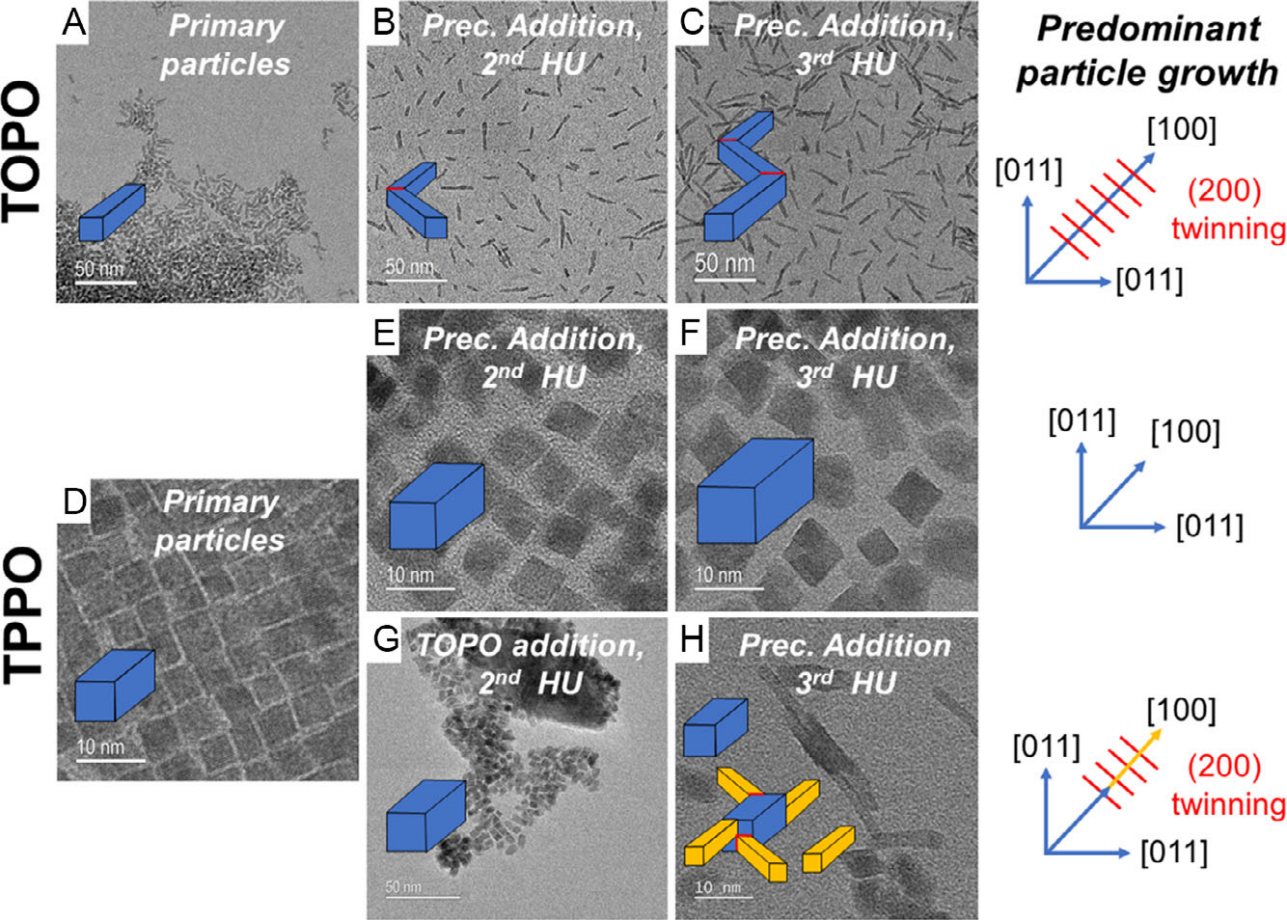
Synthesis schemes for consecutive growth. A–C) The TEM images show the increases of length of HfO_2_ nanorods, D–F) increase of volume of the HfO_2_ nanoprisms, G) formation of HfO_2_ nanohybrides (HfO_2_‐NH) comprising thicker main nanoprisms, and H) thinner nanoprism branches. Blue cuboids show schematically the evaluation of nanorods and nanoprims, while orange cuboids in (H) highlight morphologies of newly formed particle after the last synthesis step. Note: Figure (D) and Figure 1F essentially show particles from the same synthesis procedure.

Synthesis protocols for consecutive growth were developed, which allowed to synthesize nanocrystals with a broad range of controllable characteristics, such as size, shape, and high order hierarchical structures. **Figure** **2** presents three synthesis schemes for consecutive growth using different coordinating solvents and additionally highlights the corresponding crystal growth behavior. In the consecutive growth method, the standard heat‐up protocol (ramping from 180 to 340 °C with a 5 °C min^−1^ ramp after precursor addition, holding for 2 h) was performed in the first step, yielding the primary particles (Figure 2A,D). Afterwards, the reaction mixtures were allowed to quickly cool down to 180 °C by removing the heating mantle.

Either additional precursors (Figure 2B,E) or capping agents (Figure 2G) were introduced at these lower temperatures, with the intention of largely bypassing immediate nucleation. The mixture was then heated up again to 340 °C again. Finally, one last sequence including precursor addition and a heat‐up was conducted (Figure 2C,F,H). TOPO was exclusively used as solvent in the first scheme (Figure 2A–C), while TPPO was applied in the second scheme (Figure 2D–F). In the third scheme (Figure 2D,G,H), a ligand exchange in the second step was conducted by adding 2 g of TOPO (5.2 mmol, compared to 4.0 mmol of hafnium precursors in the third step). This synthesis approach differs in that regard from those in literature, that not only the alkoxide, but also the halide species of hafnium is added as precursor to the reaction mixture, while keeping the temperature comparatively low in order to prevent nucleation.^[^ ^37^ ^]^

The HfO_2_‐NR resulting from the TOPO approach were grown to 14.5 ± 5.7 nm and 21.6 ± 7.0 nm in length (i.e., [100] zone axis) after second and third addition, respectively. No noticeable changes of their width were observed between the first and subsequent steps (see Figure S4A–C, Supporting Information). Each addition of 4 mmol of hafnium precursor resulted in ≈7 nm increase of the length of the nanorods. In contrast, similar steps of addition of precursor in TPPO resulted in a size increase in all dimensions for HfO_2_‐NP (see Figure 2D,E and S4D–F, Supporting Information). The shape and facets of the nanoprisms were identified by imaging the nanoparticles along two orthogonal directions, as will be described in detail later. The particles grew by a factor of 1.2 and 1.1 after the second and the third heat‐up step, resulting an averaged value of dimensions in *<*011*>* directions of 5.9 ± 0.9 and 6.6 ± 0.8 nm, respectively. The estimated average volume of the HfO_2_‐NP approximately doubled after the second precursor addition and increased further by a factor of 1.4 after the last addition. This suggested that about 80–100% of precursor was converted onto the preexisting particles by heterogenous growth. A ligand exchange with TOPO resulted in essentially no differences in size and shape of the nanoprisms (Figure 2G). Yet, after adding precursors to the reaction mixture and taking a heat‐up step, three different types of particles were obtained: nanoprisms being similar to those obtained from the initial reaction step, emergent long fine nanorods, and hierarchical branch nanoprisms, i.e., nanohybrides (HfO_2_‐NH, synthesis HU‐TPPO⟲TOPO), being of a combination of the former two (Figure 2H). In terms of abundance, the branched nanoprisms constitute approximately 25% of the particles in the sample, while the thin HfO_2_ nanoprisms make up only 15%, see Figure S5, Supporting Information. Due to partial agglomeration of the particles, it is cumbersome to distinguish the extent of how much the HfO_2_ nanoprism morphology has transformed toward the branched particle type during the consecutive growth steps.

### 2.3 In‐Depth TEM Analysis

**Figure 3:**
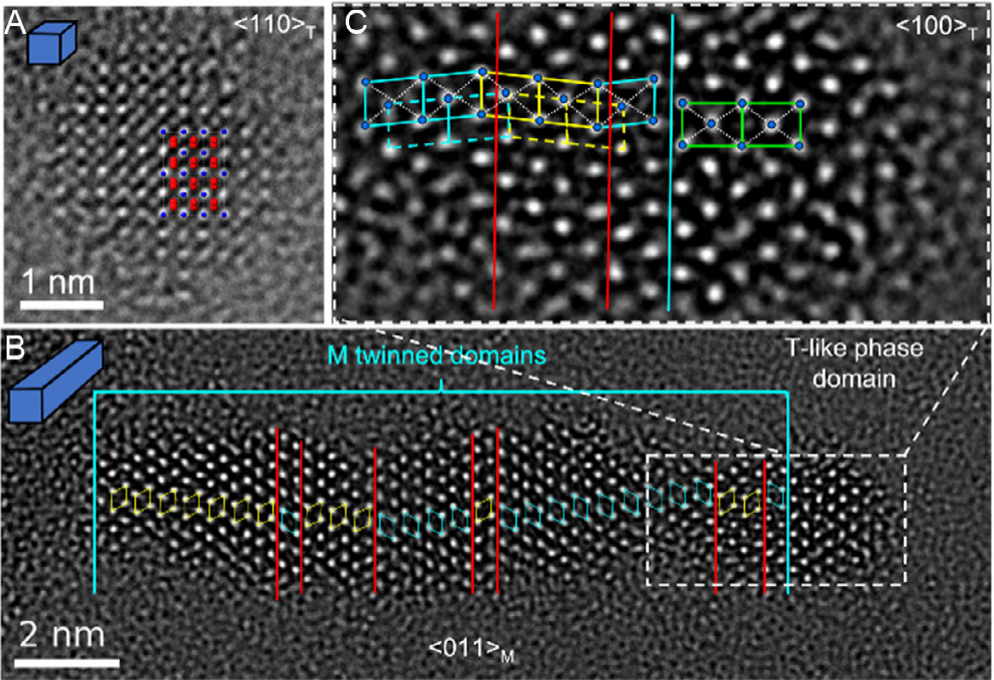
Atomic resolution NCSI TEM images of A) a tetragonal phase HfO_2_ nanodot (HI‐TOPO, see Table 1 and Figure 1A) and (B) a multiply twinned monoclinic (M) phase HfO_2_ nanorod synthesized by the consecutive growth using TOPO (see Figure 2C). Brighter dots in the NCSI images show the contrast of Hf atoms. B) Red lines indicate the {200} twin planes, which were determined by tiling of motifs that correspond to the arrangements of Hf atoms.^[^ ^42^ ^]^ C) Zoom‐in view of the right‐end region of the multiply twinned nanorods where the structure showing a centered rectangular lattice (green solid lines) appear to match with the tetragonal (T) phase projected along <100> zone axis. The structure of the monoclinic phase shows an oblique or ordinary parallelogram (cyan and yellow solid/dashed lines) lattice. The cyan and yellow obliques indicate the lattices with different orientations for which the solid and dashed lines indicated the two set of lattices corresponding to the two types of the projected Hf atom positions for the monoclinic structure. The white dotted lines indicated the diagonals of the oblique and rectangular lattices corresponding to the monoclinic and tetragonal structure to highlight the off‐center (for the former) and on‐center (for the latter) of the Hf atom column inside the lattices, respectively. The overlapping between the solid line cyan (yellow) and dashed line yellow (cyan) obliques across the twin boundary indicated by the right (left) red vertical line is resulting from the coherent nature of the twin boundary across which the two twin parts share the Hf atoms nearest neighboring to the twin boundary. In the superposed diagram of the structure, blue symbols indicate Hf atoms in (A) and (C), red for O atoms in (A).

The synthesized nanocrystals of HfO_2_ were further characterized in detail using TEM imaging methods by means of negative spherical aberration (*C*_s_) imaging (NCSI)^[^ ^42^, ^44^, ^45^ ^]^ conventional TEM (CTEM) and high‐angle annular dark‐field scanning TEM (HAADF STEM)^[^ ^46^, ^47^ ^]^ techniques. **Figure** **3**A shows an atomic resolution TEM image of a nanodots recorded using the NCSI technique oriented in its *<*110*>*_T_ zone axis. In the NCSI TEM image, Hf atoms and oxygen atoms appear as brighter and less bright dots, respectively. The observed atomic arrangement of Hf and O atoms matches well with a *<*110*>*_T_ zone axis projection of the T phase. This agrees with the result of SAED shown in Figure 1A. It should be mentioned that conclusively distinguishing between the tetragonal and cubic phases for nanocrystals with dimensions of only several nanometres remains a challenge due to their similarity. The SAEDs were recorded at a lower magnification so that the required electron dose was distributed over a much larger field of view compared to the atomic resolution images recorded via NCSI. For the latter, the as‐synthesized nanodots tended to transform from the T to the M phase under the electron beam illumination. The electron beam current was set as low as possible to minimize the electron beam irradiation induced phase transformations. Yet, this does not resolve the issue of distinguishing an as‐synthesized monoclinic phase HfO_2_ nanodot from an irradiation induced monoclinic nanodot, as the latter case cannot be completely ruled out.

Figure 3B shows a NCSI TEM image of a HfO_2_ nanorod, in which the prominent features are dominated by bright spots that correspond to the image contrast of Hf atoms. By tiling motifs of the Hf atoms,^[^ ^42^ ^]^ the major part of the HfO_2_‐NR was identified to consist of multiply twinned monoclinic structures projected along *<*011*>*_M_ zone axis direction. A number of crystal twins with domain widths of single, double, triple, and up to several unit cells were observed. The two twin domains across each twin boundary at the (200) plane are related by a two‐fold screw symmetry.^[^ ^42^ ^]^ The surface at either the bottom or the top side of the nanorod is not flat. At the right end of the nanorod the atomic structure matches better with the tetragonal phase projected along *<*100*>*_T_ direction than with the monoclinic phase of the *<*011*>*_M_ projection (Figure 3B,C). As highlighted in Figure 3C, for the tetragonal phase structure the bright spots that correspond to the projected Hf atoms form a pattern of a centered rectangular lattice indicated by the green solid lines, within which a Hf atom column is on‐center of the lattice. Whereas those for the monoclinic phase form a pattern of an oblique (or ordinary parallelogram) lattice indicated by the cyan and yellow solid lines, where an included Hf atom column is off‐center of the lattice. The color differentiates the different orientations of the oblique lattice resulting from twinning, for which the twin boundaries were indicated by vertical red lines. The oblique lattices can also be tiled using the Hf columns stand close but off‐center of the solid line obliques marked, which were indicated by the dashed lines. The overlapping between the solid line cyan (yellow) and dashed line yellow (cyan) obliques across the twin boundary indicated by the right (left) red vertical line is resulting from the coherent nature of the twin boundary across which the two twin parts share the Hf and O (not shown) atoms nearest neighboring to the twin boundary.^[^ ^42^ ^]^ We should note that the Hf atoms form also a centered rectangular (or rhombic) lattice when the cubic fluorite structure is projected along its <110> zone axis being similar to that of tetragonal structure projected along its <100> zone axis. The experimental evidence alone therefore is not sufficient to rule out one from the other. That is why we call it tetragonal‐like phase. The T‐like phase was also observed at the surfaces from other monoclinic phase HfO_2_ nanocrystals (c.f., Figure 5c and 6, as shown further below) that were synthesized in this work.

We prepared the samples to make the nanoprisms either standing up with their long axes, i.e., <100> zone axes, perpendicular to or laying down with their long axes parallel to the surface of the carbon support of the TEM grids. We imaged these samples without tilting the sample and refer the recorded images as top‐view for the former and side‐view for the latter regarding the geometry of the prism, respectively. In the case of side‐view. a considerable number of nanoprisms were projected along the [011] zone axis, but the projection of the nanoprism was not exclusively along this zone axis.

**Figure 4:**
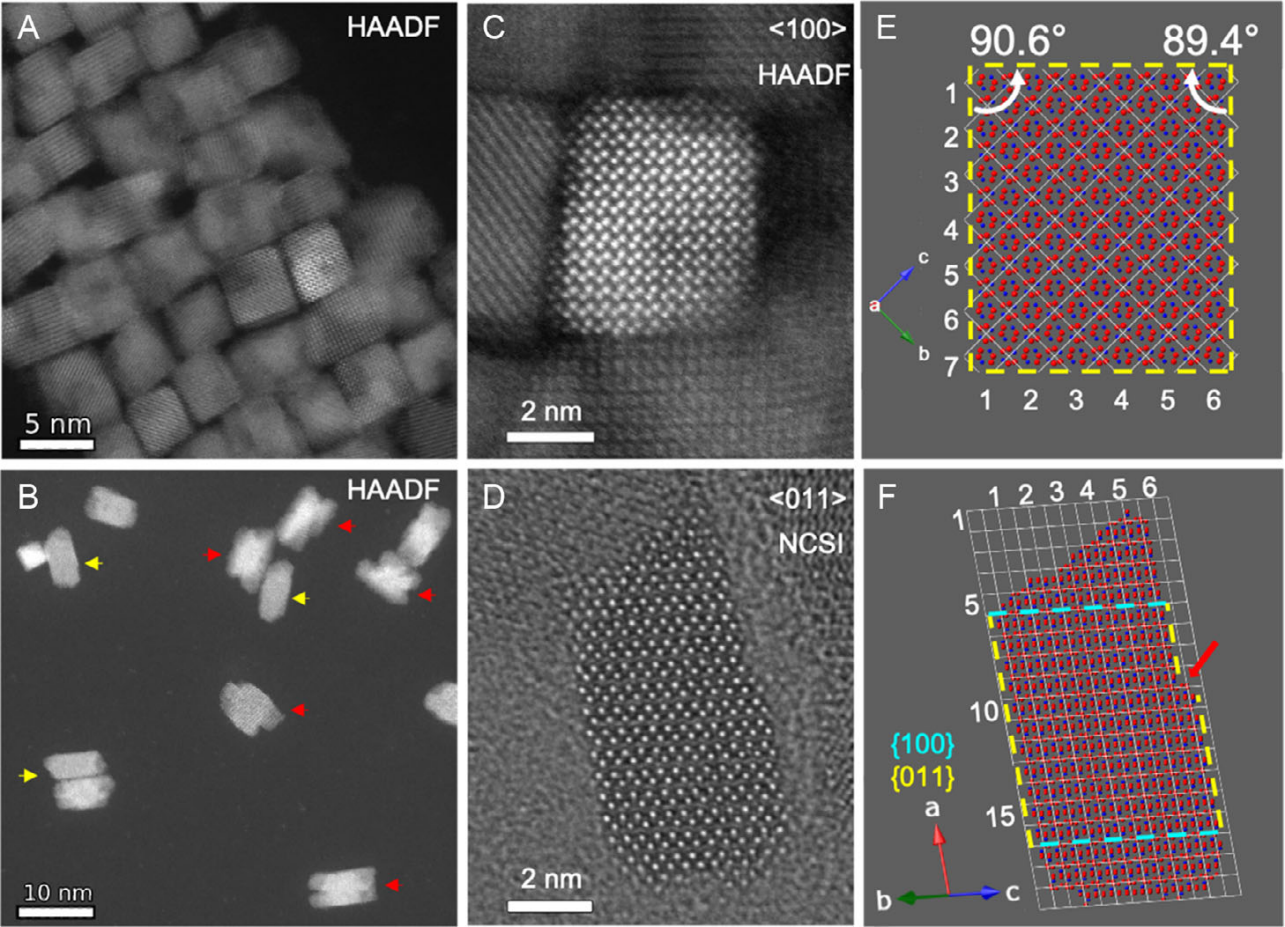
HAADF STEM images of the monoclinic phase HfO_2_ nanoprisms (sample HU‐TPPO, see Figure 1F, 2D and G,) synthesized using TPPO. A) Top‐view of nanoprisms assembled side‐by‐side. B) Side‐view of sparsely dispersed nanoprisms. Yellow and red arrows indicate nanoprisms ending with single or double tips, respectively. C) and D) show atomic resolution images using HAADF STEM and NCSI TEM techniques from nanoprisms oriented along the *<*100*>* zone axis and *<*100*>* zone axis, respectively. E) and F) structural models corresponding to (C) and (D). Blue spheres depict Hf atoms, red oxygen atoms. White lines mark the unit cells, which were indexed by numbers. Yellow and cyan colored dashed lines indicate {110} and {100} planes, respectively. The red arrow (F) marks the step observed on the left {011} surface.

The top‐view conventional TEM (CTEM) images of the nanoprisms shown in Figure 2D–G are dominated by phase contrast and hardly allow for interpretation regarding the sample thickness along the projection direction. In contrast to CTEM, the collected signal for HAADF STEM is sensitive to the atomic number (Z) of elements and has simple dependence on sample thickness, which gives rise to brighter contrast for either areas with higher Z atoms or with greater thickness in the HAADF images.^[^ ^46^, ^47^ ^]^ **Figures** **4** shows the HAADF image overview of the HfO_2_‐NP from top (A) and side (B) orientation, respectively, providing complementary information about their three‐dimensional (3D) shape. The top‐view HAADF image shows often a variation of contrast in individual nanoprisms, which indicates that the material thickness along the projection direction, i.e., *<*100*>* direction, is not uniform. The side‐view HAADF image shown in Figure 4B reveals that the surfaces at the two ends is not flat for the majority of HfO_2_‐NP, ending with single or double tips as highlighted by yellow and red arrows, respectively. This further confirms that the majority of the individual nanoprisms have nonuniform thicknesses along their long axes and is consistent with the observation in the top‐view HAADF image from Figure 4A.

Well‐defined {011} facets that parallel to their long axes were identified by atomic resolution imaging from the top and side. Figure 4C shows an atomic resolution HAADF STEM image of a HfO_2_‐NP projected along its *<*100*>* axis, in which the heavier Hf atoms appear as bright dots whereas the lighter O atoms were not resolved. The inferred atomic structural model is shown in Figure 4E, in which the surfaces at the four sides of the nanoprism agree with the {011} atomic planes as indicated by yellow dashed lines. The angles between two intersecting {011} planes are close, but not exactly 90° because of a small difference in the magnitudes projected along the *<*100*>* axis direction between the b and c unit cell vectors. The former is the same as the lattice parameter *b* (0.5168 nm) whereas the latter equals lattice parameter c times sin(*β*) (0.5222 nm). The sparsely dispersed nanoprisms laying down on the thin carbon film support of the TEM grids were often found in their *<*011*>* zone axis projection due to the flat {011} side surfaces. Figure 4D shows an atomic resolution NCSI CTEM image of a nanoprism projected along its *<*011*>* zone axis. In this case, the nanoprism corresponds to those that end with a single tip (highlighted with yellow arrows, see Figure 4B). In this image the larger brighter dots correspond to the contrast of Hf atoms and some of the O atoms were also resolved as smaller and less bright dots between the Hf atoms. A structural model deduced from the observed atomic structure is shown in Figure 4F. It can be seen that the bottom of the nanoprism is truncated and dominated by the {100} plane, but the top has a wedge shape. The surface at the left side is a flat {011} plane, whereas the surface at the right side has a step, as indicated by the red arrow, with a height of two HfO_2_ atomic layers. The observed step can act as nucleation centers and hence provides an indication of homoepitaxial growth on {011} surfaces of the monoclinic HfO_2_ nanoprism. The growth mechanism will be discussed in more detail below. While no twinning at the (200) plane was observed in this nanoprism, occasional twinning was identified HfO_2_‐NP with single tip ends.

**Figure 5:**
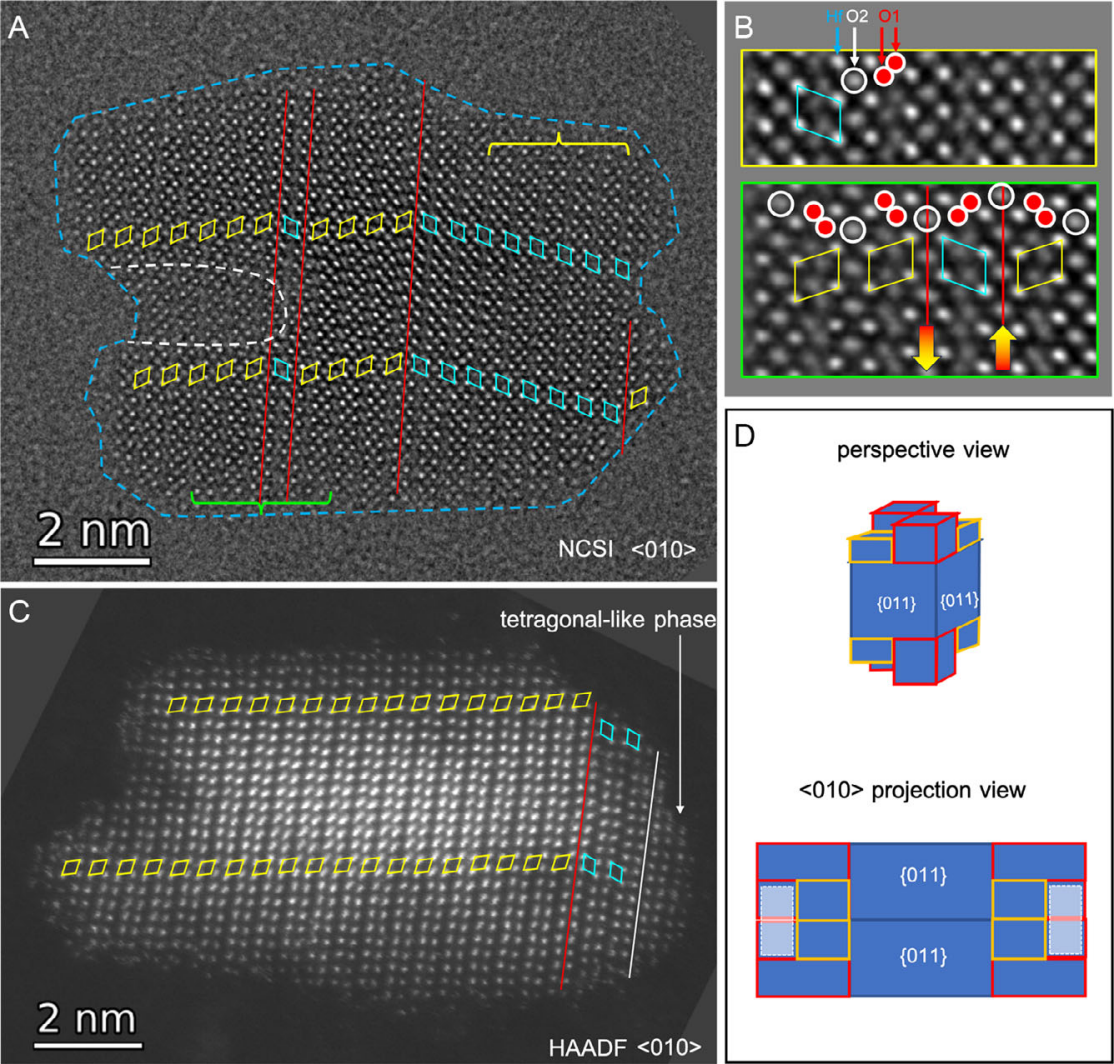
A) NCSI CTEM image of a multiply twinned nanoprisms of monoclinic HfO_2_ projected along *<*100*>* zone axis [from synthesis HU‐TPPO (3^rd^)]. Red lines indicate the (200) twin planes. The blue dashed lines indicate the outline or shape of the projection of the nanoprism. The white dashed curve at the left side indicates a thinner region reaching toward the particle center. B) Averaged images from regions of normal monoclinic structure and twin boundaries indicated by the yellow brace and green braces in (A), respectively. Averaging was performed based on the periodicity along the direction parallel to the (200) plane, which forms the twin planes indicated by red lines. The arrows filled with red‐yellow gradient point to the direction of polarization resulting from the two‐fold screw twinning at the twin boundary. C) HAADF STEM image of a nanoprism with the left end of a double tip showing a swallowtail shape. It was twinned twice close to its right end and is terminated with the tetragonal‐like phase. The location of the twin planes shown in (A) and (C) were determined by tiling of motifs that correspond to the arrangements of Hf atoms.^[^ ^42^ ^]^ D) Schematic of the shape and structure of the nanoprism disregarding *β*‐tilt of the crystal and crystal twins from a perspective (top) and *<*010*>* projection view (bottom), respectively. Here, the yellow and red edges shall emphasize on how the contrast‐loss (light blue area in the <010> projection) is created.

Using high‐resolution NCSI CTEM and HAADF STEM imaging, it was observed that when viewed along a specific projection direction near the *<*010*>* zone axis, a distinctive ‘swallow‐tail’ shape for the HfO_2_‐NP with a double tip at their ends became evident. **Figure** **5** displays a nanoprism imaged along this *<*010*>* axis (A), highlighting its double‐tipped end. It is noteworthy that the nanoprism was multiply twinned on the (200) planes, marked by red lines. Both ends of the nanoprism showcased this swallowtail shape. Images were averaged for both regular monoclinic structures and twin boundaries, specifically along the direction of the (200) plane. In the resulting images, as seen in Figure 5B, brighter, larger dots represent Hf atomic columns. Meanwhile, dimmer, larger dots indicate O columns associated with four Hf atoms, and the even fainter, smaller dots depict O atomic columns linked to three Hf atoms. By distinguishing these atomic structures, we could deduce the nature of the twinning and the resulting polarization direction.^[^ ^42^ ^]^

Further, Figure 5C demonstrates a HAADF STEM image of a nanoprism with its characteristic swallowtail shape on the left end, when oriented along the *<*010*>* zone axis. In this image, only the heavier Hf atoms are depicted as luminous dots. Certain structural features, like a twin boundary on a (200) plane and a tetragonal‐like configuration, were noticeable at specific regions of the nanoprism. Also, the nanoprism's brightness tends to increase as one approaches its central horizontal axis, indicating thicker material in that area. This brightness pattern aligns with top‐view images from Figure 4C,E, which suggest that the *<*010*>* direction aligns diagonally with the nanoprism's cross‐section. Lastly, the 3D representation of the nanoprism, with its swallow‐tail shape when projected in the *<*010*>* orientation, is graphically illustrated in Figure 5D.

**Figure 6:**
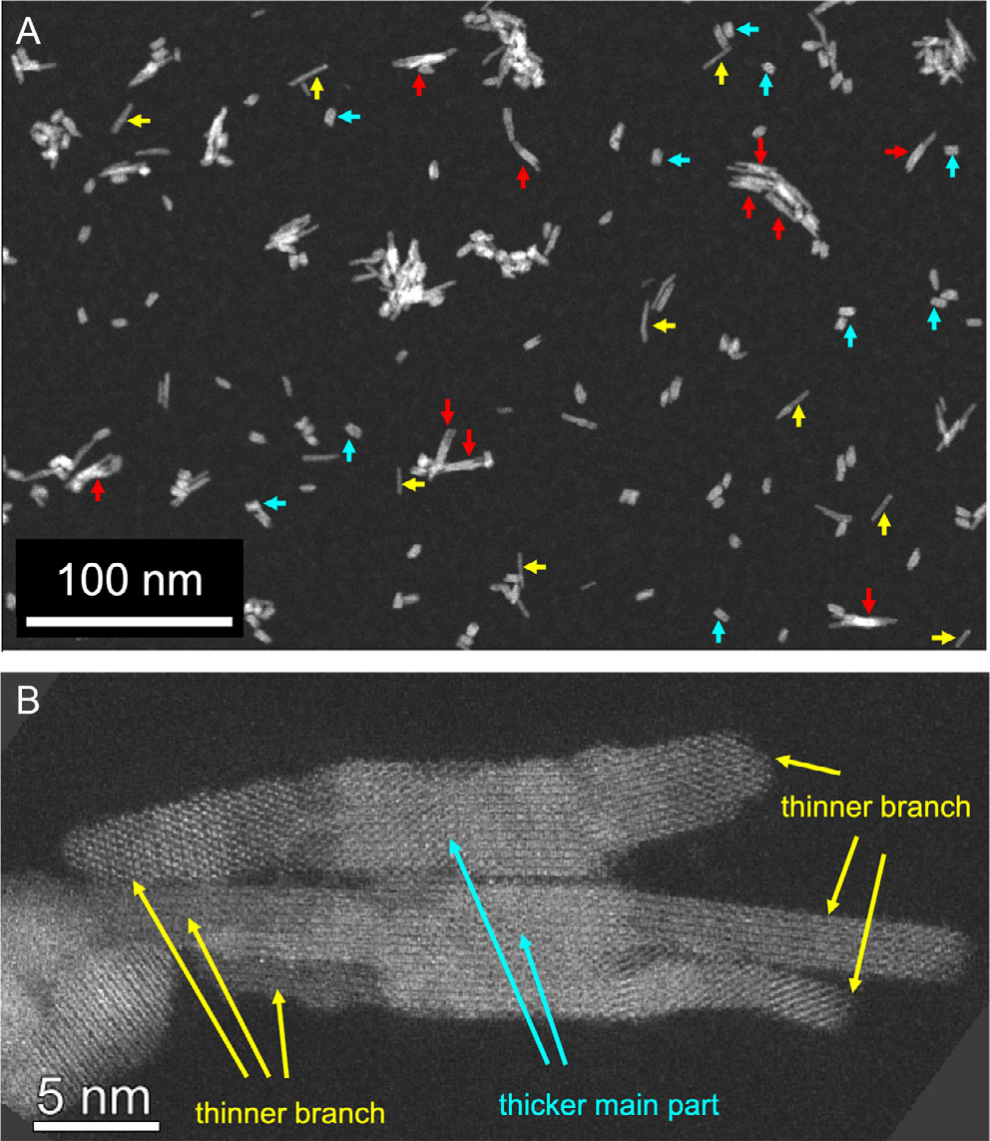
A) Overview HAADF STEM image of HfO_2_‐NH (from synthesis HU‐TPPO⟲TOPO) with long fine nanorod branches that are indicated by red arrows. Individual short thick nanoprisms and long fine nanoprisms are indicated by yellow and cyan colored arrows, respectively. B) High resolution HAADF STEM image of two HfO_2_‐NH with long and finely‐branched nanorods viewed along a direction close to *<*011*>*.

**Figure** **6**A presents a comprehensive HAADF STEM image of a HfO_2_ nanohybride sample. Within this image, nanoprisms featuring elongated fine branches are highlighted with red arrows. The actual count of these branched nanoprisms in the image might be underestimated since the agglomerated particles are not considered. Besides, the sample also contains individual thick, short nanoprisms and elongated, thin nanorods. These particle classes were previously mentioned in the consecutive growth section. A closer look is provided in Figure 6B, which displays a high‐resolution HAADF STEM image of two HfO_2_–NH, both of which have elongated fine branches. From this image, one can discern that the primary HfO_2_–NP parts in the center of the particle are wider, brighter, and consequently thicker. Conversely, the branched rod‐like sections appear narrower, dimmer, and therefore thinner.

### 2.4 Self‐Assembly Behavior

**Figure 7:**
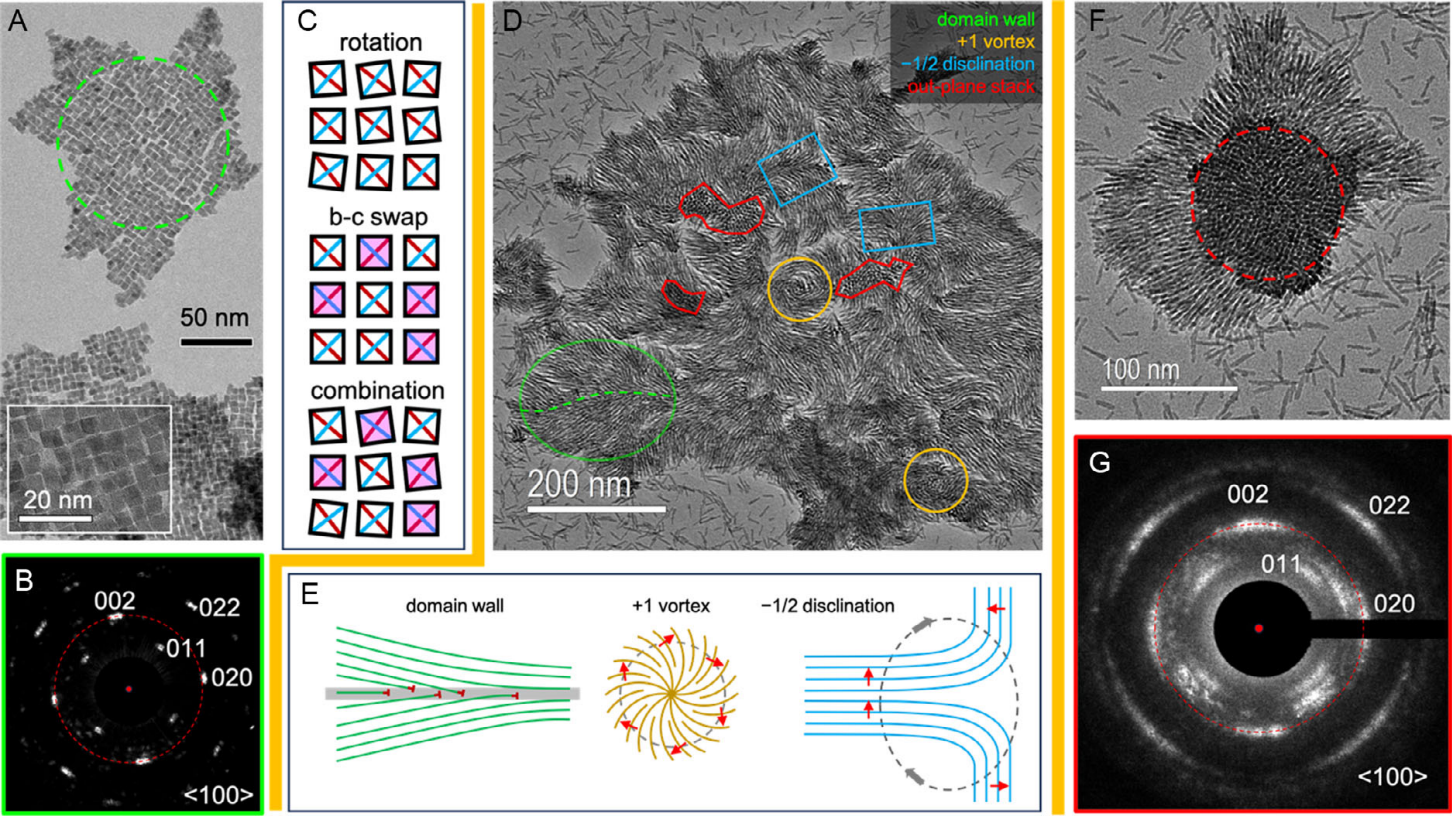
A) Conventional TEM (CTEM) image of self‐assembly of short thick nanoprisms of HfO_2_. The inset shows a high magnification image. B) Selected area electron diffractogram from an area indicated by the dashed circle in (A). C) Schematic diagram of in‐plane orientation distortions in self‐assemblies of HfO_2_ nanoprisms resulting from rotation by small angles (top), a swap of *b*‐*c* axes (middle), and their combination (bottom). Black squares denote the {011} surfaces of the nanoprisms. The two diagonals indicated by red and blue lines denote the crystallographic *b*‐ and *c*‐axis, respectively. The shadow highlights the swap of *b*‐*c* axes. D) CTEM image of self‐assembly of HfO_2_ nanorods, in which a domain wall, vortices, disclinations, and out‐plane stacks were marked. E) Schematic diagram of the structure of the domain wall, vortices, and disclinations observed in (D). The topological charge or strength for the vortex and the disclination is +1 and −1*/*2, respectively. Long axes of the nanorods were aligned along solid lines. Red arrows indicate the normal of the lines. F) CTEM image of self‐assembly of a bunch of HfO_2_ nanorods with their axes normal to the support, i.e., the out‐of‐plane alignment. G) SAEDP from the area indicated by the red dashed circle in (F). In (B) and (G), the red dot marks the center of the diffraction pattern. The red dashed circle indicates the length of the diffraction vector of the (002) planes.

The formation of self‐assembled structures on the thin carbon support of TEM grids was observed for both HfO_2_‐NP (primary and consecutively grown) and the long HfO_2_‐NR from the consecutive growth method [sample HU‐TOPO(3^rd^)]. The HfO_2_‐NP tend to self‐assemble in monolayers forming a pseudo‐square 2D lattice (**Figure** 2D and **7**A and inset). In the assembly, each nanoprism is nearest neighboring to four other nanoparticles with their {011} facets. Figure 7B shows a selected area electron diffractogram recorded from the area marked by the green dashed circle in Figure 7A. It can be seen that the diffraction spots show a noticeable angular spread. This can be explained by the presence of in‐plane small angle rotational distortions (Figure 7C). The diffraction spots resulting from the (002) planes with d‐spacing of 0.261 nm are on the red circle whereas the (020) diffraction spots are just outside of the circle due to a slightly smaller *d*‐spacing of 0.258 nm for the (020) planes. This implies that the occurrence of *b*‐*c* axes swaps is negligible because a random swap of the *b*‐*c* axes of the nanoprisms will cause a radial spread of the (020) and (002) diffraction spots making them indistinguishable. A noticeable angular spread of the diffraction spots suggests that the in‐plane orientation distortions are dominated by small angle rotation.

Structures observed in the self‐assembly of the long thin nanorods (Figure 7D) are remarkably reminiscent of the characteristic feature of nematic liquid crystals that are made up of rod‐like molecules.^[^ ^48^, ^49^, ^50^ ^]^ The majority of long thin HfO_2_‐NR were laying down on the support and some of them formed out‐of‐plane stacks. A domain wall and topological defects of vortices and disclinations were observed in the self‐assembly. Their structures are schematically illustrated in Figure 7E. The observed domain wall separates two adjacent domains that are tilted relative to each other by a small angle. A great number of dislocations were accumulated at the domain wall. The structure of domain wall is similar to that of smectic‐A liquid crystals described by Pershan in ref. [49].

Vortices and disclinations are well‐known topological defects observed in nematic liquid crystals.^[^ ^48^, ^50^ ^]^ They can be labelled by their winding number, also known as the topological charge or strength, which equals 2*π/*Ω being an integer or a half integer number.^[^ ^50^ ^]^ Here Ω is the angle in radians that the local director rotates when traveling around the defect. In Figure 7E, the red arrow indicates the normal of the local director, i.e., the long axis of the nanorod. The topological charge is positive when the direction of the local director rotates in the same sense as the traveling and vice versa.^[^ ^51^ ^]^ The topological strength of observed vortexes and disclinations were thus identified to be +1 and −1*/*2, respectively where the −1*/*2 disclination is half of a − 1 disclination.

Figure 7F shows a cluster of long thin HfO_2_–NR with their long axes normal to the support. The nanorods laying down on the support were concentrically and radially aligned around the out‐of‐plane stack. The SAEDP from the out‐of‐plane stack is shown in Figure 7G. Although the stand‐alone (002) and (020) diffraction spots can still be distinguished, the diffraction spots have considerable radial and angular spread. The stand‐alone diffraction spots instead of diffraction rings suggests the long thin nanorods are likely non‐straight prisms. For a random in‐plane crystallographic orientation of genuine HfO_2_–NR an overlapped diffraction ring of (002) and (020) rather than separated diffraction spots are expected (compare with SAED from Figure 1B–E). While the considerable radial and angular spread of the diffraction spots implies appreciable in‐plane orientation distortions resulting from a combination of small angle rotation and *b*‐*c* axis swap (Figure 7C). This can be a consequence of the twinning and nonuniform widths that have been described for HfO_2_–NR above (Figure 3B).

## 3 Discussion

### 3.1 Crystal Growth Mechanism

Due to the preference for HfO_2_ to form thin nanoprisms even with minute amounts of TOPO in the reaction mixture, we conclude that TOPO has a stronger surface binding affinity toward hafnia surfaces. Similar effects on crystal morphology formation discrepancies have been found for semiconductor nanocrystals that were synthesized with deliberately added impurities of phosphonic acids to TOPO.^[^ ^52^, ^53^ ^]^ This is in line with the fundamental understanding of surface acidic metal centers that bind basic species. In this case, the Hf(IV)‐cation, i.e., a strong acid, shows a more pronounced affinity toward the P=O headgroup of TOPO, which constitutes a strong base. Oxygen reduction analyses reveal, that the oxygen lone pair in phosphine oxides with alkyl substituents more readily form bonds compared to aryl substituents.^[^ ^54^ ^]^ We therefore assume that TOPO likewise acts as a stronger base when compared to TPPO, likewise. However, one needs to transfer these assumptions to the actual surface binding chemistry, which in literature was described as decomposition products of the phosphine oxides (i.e., phosphinic‐ and phosphonic acids).^[^ ^55^ ^]^

Furthermore the results give evidence that a hot‐injection approach can indeed be used to stabilize HfO_2_ nanoparticles in the T phase and a quasi‐spherical morphology. This holds true despite the fact that the size distribution is not necessarily below the earlier discussed critical size threshold of 3.6 nm.^[^ ^13^, ^39^ ^]^ It should be emphasized that the difference in outcome between samples HI‐TOPO and HU‐TOPO, i.e., varying the heating protocol, was reproducible. To unambiguously attribute the T phase formation to the HI‐method, an analogue experiment was also conducted at 390 °C, which yielded rodlike particles in a HU approach according to literature (see Figure S7, Supporting Information).^[^ ^35^ ^]^ The product from the 390 °C HI‐experiment consisted of T phase HfO_2_ nanocrystals that showcased an even smaller size distribution compared to the sample HI‐TOPO. Hence, the experiments elucidate the contradictory descriptions of why either M HfO_2_–NR or T HfO_2_ nanodots could be synthesized under seemingly similar conditions in the past: a rapid heating ramp can effectively be understood as hot‐injection approach. We propose that the underlying mechanism is that at higher temperatures, the role of surface energy biases are diminished, regardless of whether they arise from selective surface adherence or preferred growth of high‐energy facets. In such conditions, the dynamic adsorption and desorption of surface‐bound molecules permit greater nucleation at random crystal facets and a general nucleation of seeds in solution. As a consequence, existing nuclei will reduce their surface energy by developing an isotropic morphology. The quasi spherical particles from the HI approach thereby display the equilibrium shape of the nanocrystals that stay in T phase with reduced shear strain due to lower anisotropy. Very recent findings on this non‐aqueous synthesis goes even further and has shed light on the crystallization mechanism that suggests an amorphous intermediate phase which precedes crystallization. In this study, zirconium oxide nanocrystals have uncovered such amorphous intermediates, which are likely to be a fundamental aspect of their synthesis pathway. Given the chemical and physical similarities between zirconium and hafnium, we surmise that our hafnium oxide nanocrystals may also progress through a similar amorphous phase. This is consistent with our observations of amorphous‐like material at the tip ends of the nanorods.^[^ ^38^ ^]^

Based on these results we hypothesize that the divergence between the HU and the HI approach is associated with nucleation kinetics in the reaction medium. Further recent findings in literature detail an investigation into the disparities in the context of this sol‐gel synthesis.^[^ ^40^ ^]^ The authors’ focus was mainly set on zirconium precursors, with a strong emphasis on the role of precursor molecular chemistry, but they also transferred their findings toward hafnium. According to their study, the active species, HfCl_2_(O*i*Pr)_2_)·2TOPO, which is a product of Hf(−O*i*Pr)_4_, HfCl_4_ and TOPO, was found to go through a sequence of reactions, leading to the creation of hafnia monomers. The authors observed that the active HfCl_2_(O*i*Pr)_2_)·2TOPO concentration decreases at 200 °C, while the HfCl_3_(O*i*Pr)·2TOPO concentration increases, suggesting that the reaction is largely completed by the time the temperature reaches 360 °C. It is inferred that the nucleation process for HfO_2_ occurs earlier in the heating ramp, specifically between 200 and 340 °C, which highlights the significance of the heating process in the synthesis.

Nucleation theory and crystal growth dynamics offer insights into the bias for nuclei to grow longitudinally in a HU approach.^[^ ^56^, ^57^ ^]^ The hafnium precursor mixture in the HU method generates potential active monomers, and nucleation likely commences within the 200–340 °C temperature range, leading to a kinetic growth regime.^[^ ^58^ ^]^ The role of facets in crystal growth is pivotal, with {100}‐like high‐energy facets initiating growth along the *a*‐axis of the crystal, whereas low‐energy facets resist such growth.

This emphasizes the phenomenon of “selective crystal surface adherence” where organic molecules preferentially bind to certain crystal surfaces, restricting homoepitaxial growth in directions other than the [100] orientation. Moreover, already existing particle moieties can also serve as potential nucleation sites and therefor catalyze the decomposition of the precursors and lead to accelerated particle growth.^[^ ^59^ ^]^ “Oriented attachment” is another process in focus for HfO_2_, involving a unique type of coalescence that diminishes surface energy in the system and is often associated with twinning in nanocrystals.^[^ ^60^ ^]^

In the following we therefore contrast the homoepitaxial nucleation and growth mechanism with that of the oriented attachment process in accordance to our findings: 1) Although the merging of smaller nuclei along the {100} direction might elucidate the directional twinning, the unit‐cell level twinning seen in both, HfO_2_‐NR and HfO_2_‐NP, challenges this notion. Furthermore, the occurrence of T‐like or amorphous phases at tip ends also indicate epitaxial nucleation and growth alongside the *<*100*>* axis.^[^ ^42^ ^]^ 2) The step defect (see Figure 4) can be considered as an example for a typical nucleation center for layer‐by‐layer homoepitaxial growth on {011} surfaces.^[^ ^61^ ^]^ 3) The size distribution for the consecutive growing mechanism is increased, yet broadened after each synthesis step. In an oriented attachment scenario, a more discrete size distribution should be anticipated. and 4) The emergence of swallow‐tailed tip ends in thicker HfO_2_‐NP is atypical and does not readily align with a continuous growth mechanism. Meanwhile, four side‐by‐side attached HfO_2_‐NR could explain the formation of such a morphology. However, the abundance of organic capping agents that would need to be stripped off the {011} facets during that process in order to support this hypothesis. TPPO appears to favor this because it is prone to desorb more easily from the {011} facets as shall be discussed later.

**Figure 8:**
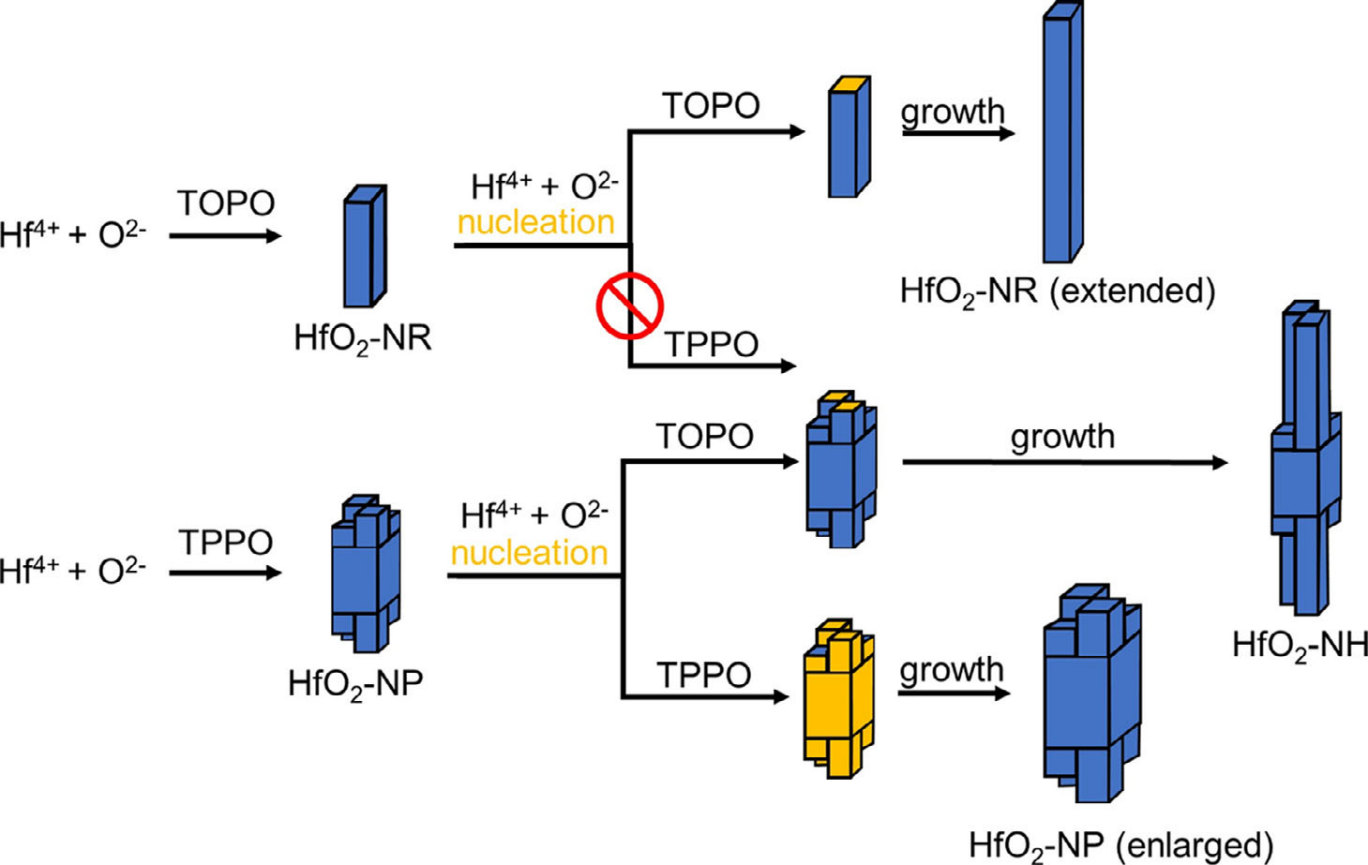
Formation process of consecutively grown HfO_2_ nanoparticle morphologies.

Hence, the evidence more strongly supports the homoepitaxial nucleation and growth mechanism over the oriented attachment process. We therefore summarized our hypothesis for this consecutive growing mechanism in **Figure** **8**.

However, the abundance of organic capping agents that would need to be stripped off the {011} facets during that process in order to support this hypothesis. TPPO appears to favor this because it is prone to desorb more easily from the {011} facets as shall be discussed later. Hence, the evidence more strongly supports the homoepitaxial nucleation and growth mechanism over the oriented attachment process. We therefore summarized our hypothesis for this consecutive growing mechanism in Figure 8.

The swallow‐tail *a*‐axis terminations in the TPPO‐based HfO_2_–NP can be understood as layout for the branched HfO_2_‐NH. We assume that the tetrapodal structure of the particles results from 2 × 2 columnar growth, where only two diagonally opposing columns can be accessed by HfO_2_ monomers and subsequent growth. For that matter, TOPO enables consecutive growth only at {100}‐like surfaces, while for TPPO the growth is only inhibited at {100}‐like terminations at columns with reduced lengths, i.e., space‐limited columns. The nucleation mechanism can be two‐fold: 1) the TOPO‐stabilized monomers only have access toward {100}‐like terminations through space hindrance, because the {011}‐like facets are densely covered by TOPO ligands that feature long hydrocarbons chains. and 2) As it was already discussed, TOPO is assumed to bind stronger to the HfO_2_ surfaces and thereby is less likely to desorb from the {011} facets. TPPO, in contrast, seems to allow *<*011*>* directional growth, which suggests that it is prone to desorb more easily. Although it is not necessarily the case, the columnar extensions feature twinning.

Usually only one of the opposing columns shows such a twin boundary, which probably results from preventing a dislocation defect within the *a*‐axis termination (see examples in Figure S8, Supporting Information).

### 3.2 Wulff‐Construction Analysis of Monoclinic HfO_2_

**Figure 9:**
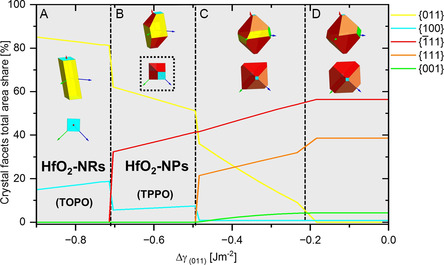
Simulated Wulff‐construction area share of each facet which is plotted against the change of Δ*γ*_(011)_. Specific energy regions are separated with dashed lines and indicated as from A‐D with corresponding simulations of the crystal morphology (one from a general crystal projection, one from the *<*100*>* zone‐axis). All Wulff‐construction analyses have been performed with the WulffMaker Software with *γ* parameters based on the DFT calculations by Mukhopadhyay et al.^[^ ^85^, ^86^ ^]^

Interestingly, when one applies the Wulff‐construction method on the stabilization of the {011} surfaces (i.e., deliberately lowering *γ*_(011)_ while fixing all other *γ* values that were retrieved from literature, see Table S2, Supporting Information), the respective motifs are in accordance with the TOPO‐stabilized HfO_2_–NR and the TPPO‐stabilized HfO_2_–NP, see **Figure** **9**.^[^ ^54^, ^55^ ^]^

The Δ*γ*_011_ regime in D shows that the crystal mainly consists of the low‐energy $\left\{\bar{1}11\right\}$‐ and {111} facets, just less than 10% of {001} and {100} facets exhibit no {011} faceting. When Δ*γ*_011_ decreases to −0.49 Jm^*−*2^, the area shares of {011} facets now appears and increases to 35 % (Δ*γ*_011_ regime C). Yet, for the Δ*γ*_011_ energy windows of C and D, we could not observe corresponding particle morphologies in TEM. This changes in the Δ*γ*_011_ regime of B, where the Wulff‐construction shows a particle morphology that has similarities with the TPPO‐stabilized HfO_2_‐NP with only one tip end. Especially when the Wulff‐construction is depicted from the *<*100*>* zone axis (black‐dotted rectangle) the differences in thickness generate a 2 × 2 checkerboard pattern which is somewhat reminiscent of the thickness contrast from the HAADF image (see Figure 4C) recorded from the same zone‐axis. If Δ*γ*_011_ is reduced beyond −0.71 Jm^*−*2^ (D), presumably the particle only consists of {011} and {100} facets. Despite the fact that a clean {100} high‐energy facet termination cannot be observed in any of the particles, the resemblance of the Wulff‐construction of thin TOPO‐stabilized HfO_2_‐NR is striking. Analogue to the {011} facet, we generated a similar plot for the undetected {001} facets and varied *γ*_001_ correspondingly (see Figure S9, Supporting Information), but the received morphologies were not detected in TEM.

We tentatively conclude that the Wulff‐constructions in Δ*γ*_011_ A and B outline the particle blueprint for subsequent growth. However, for thicker HfO_2_ nanoprisms, growth complexity increases due to multiple nucleation surfaces and twinning. Furthermore, the Wulff‐construction does not account for the formation of swallow‐tail tip ends. In summary, for HfO_2_ consecutive growth, the variations stem from facet adherence changes between TOPO and TPPO, amplifying Δ*γ*_011_, leading to two distinct particle designs and thus a diffusion‐limited epitaxial growth mechanism has to be anticipated.

### 3.3 Topological Defect Features in HfO_2_ Self‐Assemblies

Assemblies of a square 2D lattice were observed from nanocubes of metals,^[^ ^62^, ^63^, ^64^, ^65^ ^]^ chalcogenides,^[^ ^66^, ^67^ ^]^ halides,^[^ ^68^ ^]^ and oxides,^[^ ^69^, ^70^, ^71^, ^72^, ^73^ ^]^ whose crystallographic structure is cubic. Such kind of assemblies are rarely reported for nanocrystals of monoclinic materials because the low symmetry of the crystal structure of the material makes it difficult to grow nanocrystals with well‐defined facets. The self‐assembly of nanoprisms of monoclinic HfO_2_ (Figure 7A) reported in this work represents a remarkable example of this possibility, and hence the nanoprisms of monoclinic HfO_2_ with flat {011} facets enrich the choice of nanocrystals for self‐assemblies. The self‐assembly of long thin nanorods of HfO_2_ and its topological defects including domain walls, vortexes, and disclinations (Figure 7D,E), provide opportunities to study the collective effects on the dielectric, electronic, and mechanical properties.^[^ ^74^, ^75^ ^]^ The resemblance between the self‐assembly and nematic liquid crystals implies that the long thin nanorods of HfO_2_ could be used as an additive to conventional nematic liquid crystals and those made of nanorods of other materials to form hybrid materials without significantly altering the structure. Because the twinning in nanorods of HfO_2_ (Figure 3B) results in polarization and provides a mechanism to accommodating deformation,^[^ ^42^, ^76^ ^]^ and also because bulk HfO_2_ has a high dielectric constant of *ε* = 18–40,^[^ ^77^, ^78^, ^79^ ^]^ the proposed hybrid materials may possess improved and emerging of new dielectric, electro‐optical, and mechanical properties. For example, the long thin nanorods of HfO_2_ could be mixed with these nanorods of semiconductors (e.g., CdS, CdSe)^[^ ^74^, ^80^, ^81^ ^]^ and metals (e.g., Au, Ag)^[^ ^82^, ^83^ ^]^ to form hybrid nematic liquid crystals of nanorods. On the one hand, optoelectronic properties of devices could be tuned by hybridization between conventional or semiconductor nanorods nematic liquid crystals and HfO_2_ nanorods because the local electric field will be altered in a model of series capacitors of different capacities (dielectric constant). On the other hand, as surface plasmon resonances from the metal nanorods are related to the dielectric constant of the surrounding medium.^[^ ^84^ ^]^ Modified surface plasmonic properties can thus be expected from hybrid nematic liquid crystals of HfO_2_ and Au nanorods due to a much higher dielectric constant of HfO_2_ than otherwise normal materials being used to make nanorods for hybridization.

## 4 Conclusion

We introduced a new reaction protocol that facilitates site tailoring and crystal surface engineering of colloidal hafnia. A hot‐injection method resolved longstanding contradictions regarding the properties and formation of tetragonal HfO_2_ nanocrystals and monoclinic HfO_2_–NR. Moreover, we combined in the well‐known TOPO approach TPPO as a counteracting solvent and surface stabilizer. This protocol prompts nanocrystal growth in a quasi‐epitaxial manner, unveiling novel HfO_2_ nanoprisms with atomically flat {011} surfaces. Such advancements not only refine the synthesis approach for HfO_2_ nanoparticles in general, but also revealed new insight into the mechanism of particles formation and growth and it disclosed new pathways to achieve anisotropic particles, partially with unusual shape. Thereby, a unique feature is the deliberate synthesis of nanoparticles that fuse both HfO_2_ nanorods and nanoprisms, producing branched nanohybrides.

## 5 Experimental Section

5.1

5.1.1

##### Synthesis and Purification of HfO_2_ Nanocrystals

The general synthesis approach for HfO_2_ nanocrystals was adopted from Tang et al.^[^ ^31^ ^]^ Acetone (99.8%, VWR), toluene (≥99.5%, VWR) and methanol (99,9%, ThermoFisher Scientific) were used as received for purification of the nanocrystals. The precursors hafnium (IV) chloride (99.9%, ABCR) and hafnium (IV) isopropoxide (99%, Alfa Aesar) were used without further purification. The capping agents TOPO (98%, Alfa Aesar) and TPPO (99%, ThermoFisher Scientific) were degassed to 1 × 10^−3^ mbar and flushed with argon several times. They were kept under vacuum at either 120 °C for TOPO, or at 160 °C for TPPO and TOPO/TPPO mixtures for 1 h prior to use. All syntheses were performed under argon atmosphere in a 100 mL three‐necked round‐bottom flask. Equimolar amounts of precursor were used throughout this work (2 mmol, 0.64 g HfCl_4_ and 2 mmol, 0.95 g hafnium (IV) isopropoxide). Both, hafnium (IV) isopropoxide and ‐chloride were mixed in a glass transfer device prior to addition to the reaction mixture in an argon‐filled glove box. This ensures the homogeneity of the precursors before their introduction against an argon stream to the reaction vessel.

In deviation from previously described experiments, two specific heating protocols were used for the synthesis of HfO_2_ nanocrystals. For the temperature gradient‐based method, the precursors were added to the melted capping agents (i.e., TOPO, TPPO, or TOPO/TPPO mixture) at 180 °C. While vigorous stirring, the reaction mixture was slowly heated up to 200 °C and subsequently the temperature was increased in 5 K min^−1^ steps to 340 °C, kept at that temperature for 2 h and was afterwards cooled down to 80 °C (or to 180 °C for repeated precursor addition). For the hot‐injection like approach, the powderous precursor mixture is cast into the 340 °C hot solvent against argon flow. The technique and glassware that is used during this step shown in Figure S10, Supporting Information.

The clean‐up was performed through several washing and centrifugation cycles. Acetone or methanol were used as washing and redispersing solvent for TOPO or TPPO and TOPO/TPPO respectively. The surface functionalized particles were collected as precipitate through centrifugation for 15 min at 12 000 rpm and were readily dispersable in toluene or methanol. The dispersions of TOPO‐synthesized particles were stable for several days, while the particles synthesized from TPPO sedimented within a couple of hours.

##### TEM

The HfO_2_ nanocrystals dispersed in toluene or dietethyl ether were drop‐casted onto a carbon coated copper grid (200 mesh, 3 and 4 nm, Science Services) for TEM characterization. Particle size, shape, and crystal phase were determined with standard bright field imaging techniques and SAED by TEM on a ZEISS LIBRA 200 FE microscope operated at 200 keV. Negative spherical aberration corrected imaging (NCSI) was performed at 300 kV on a FEI Titan 80‐300 and a Thermo Fisher Scientific Spectra 300 microscopes. The former is equipped with a field emission gun (FEG), a CEOS CETCOR C_S_ corrector for the objective lens, and a Peltier cooled Gatan Ultrascan 1000 P charge coupled device camera (CCD). The latter is with a high‐brightness X‐FEG monochromated source, a piezo‐enhanced CompuStage, a S‐CORR *C*_S_ corrector for the condenser lens, a CEOS CETCOR *C*_S_ corrector for the S‐TWIN objective lens, a fast Ceta CMOS camera, and a Fischione Model 3000 HAADF detector. HAADF STEM imaging was performed at 200 kV with the beam convergence semi‐angle of 20–25 mrad and detector inner collection semiangle about 60 mrad on the Spectra 300 microscope and on a FEI Titan G2 80‐200 ChemiSTEM microscope with a high‐brightness X‐FEG, a CEOS DCOR C_S_ corrector for the condenser lens, and a Fischione Model 3000 HAADF detector. Twinning in monoclinic phase HfO_2_ were identified by a tiling procedure in the TEM images.^[^ ^42^ ^]^

## Conflict of Interest

The authors declare no conflict of interest.

## Supporting information

Supplementary Material

## Data Availability

The data that support the findings of this study are available from the corresponding author upon reasonable request.
