# Supplementary material for: Tailoring of Colloidal HfO2 Nanocrystals with Unique Morphologies and New Self‐Assembly Features
Source: Small Sci. 2024 Jan 24;4(4):2300209. doi: 10.1002/smsc.202300209 (PMC11935035; doi:10.1002/smsc.202300209)
Supplement: Supplementary file 1 — Supplementary Material [file SMSC-4-2300209-s001.pdf]

Supporting Information

Tailoring of Colloidal HfO_2 Nanocrystals with Unique Morphologies and New Self-Assembly Features

Thorsten Ohlerth, Hongchu Du, Thomas Hammoor, Joachim Mayer, Ulrich Simon**

Dr. T. Ohlerth, T. Hammoor, Prof. U. Simon
Institute for Inorganic Chemistry RWTH and JARA - Fundamentals of Future Information Technologies, 52074 Aachen, Germany
Email Address: ulrich.simon@ac.rwth-aachen.de

Dr. H. Du, Prof. J. Mayer
Ernst Ruska-Centre for Microscopy and Spectroscopy with Electrons, Forschungszentrum Jülich GmbH, Central Facility for Electron Microscopy, RWTH Aachen University and JARA - Fundamentals of Future Information Technologies, 52425 Jülich, Germany
Email Address: h.du@fz-juelich.de

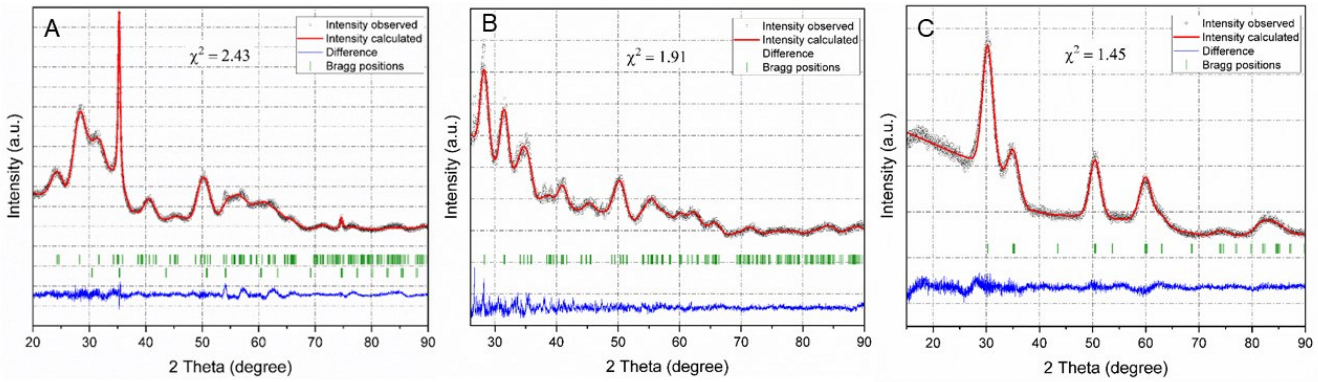

Figure S1: XRD diffractograms with Rietveld refinements of HU-TOPO(3rd) (monoclinic HfO_2-nanorods, (A), HU-TPPO (monoclinic HfO_2-nanoprisms, B) and HI-TOPO (tetragonal HfO_2-nanocrystals, C). For the nanorods, both the structural refinements of all compounds were carried out by the Rietveld method using FullProf (χ^2 values are given in each graph). Peak profiles were fitted with pseudo-Voigt functions. The atomic occupancy and isotropic atomic displacement factors of all elements were fixed. The patterns were refined for lattice parameters, scale factor, background, atomic coordinates and pseudo-Voight profile function (U, V, W, X) and preferred orientation (sample A for the [100] orientation of the nanorods). The calculated atomic positions and cell parameters of monoclinic as well as tetragonal phases are in good agreement with the experimental findings (Table S1). Please note that in order to increase the refinement quality, for the nanorods both, the monoclinic and tetragonal phases had to be included in the refinement process, which we estimate reasonable due to the occurrence of tetragonal phases at the tip ends of the nanorods.

Table S1: Calculated atomic positions and cell parameters of monoclinic as well as tetragonal phases from Rietveld refinement.

Sample	Phase	Space group	Lattice parameters [Å]		Fraction [%]
			Theoretical	Experimental	
**A**	Monoclinic	$P2_1/c$	a = 5.11, b = 5.17, c = 5.30, and β = 99.180 ICSD-27313	a = 5.14, b = 5.18, c = 5.31, and β = 99.658	99.15
	Tetragonal	P 42/n m c	a = b = 3.58, c = 5.19 ICSD-173966	a = b = 3.59, c = 5.09	0.85
**B**	Monoclinic	$P2_1/c$	a = 5.11, b = 5.17, c = 5.30, and β = 99.180 ICSD-27313	a = 5.15, b = 5.17, c = 5.33, and β = 99.174	100
**C**	Tetragonal	P 42/n m c	a = b = 3.65, c = 5.33 ICSD-7146	a = b = 3.63, c = 5.09	100

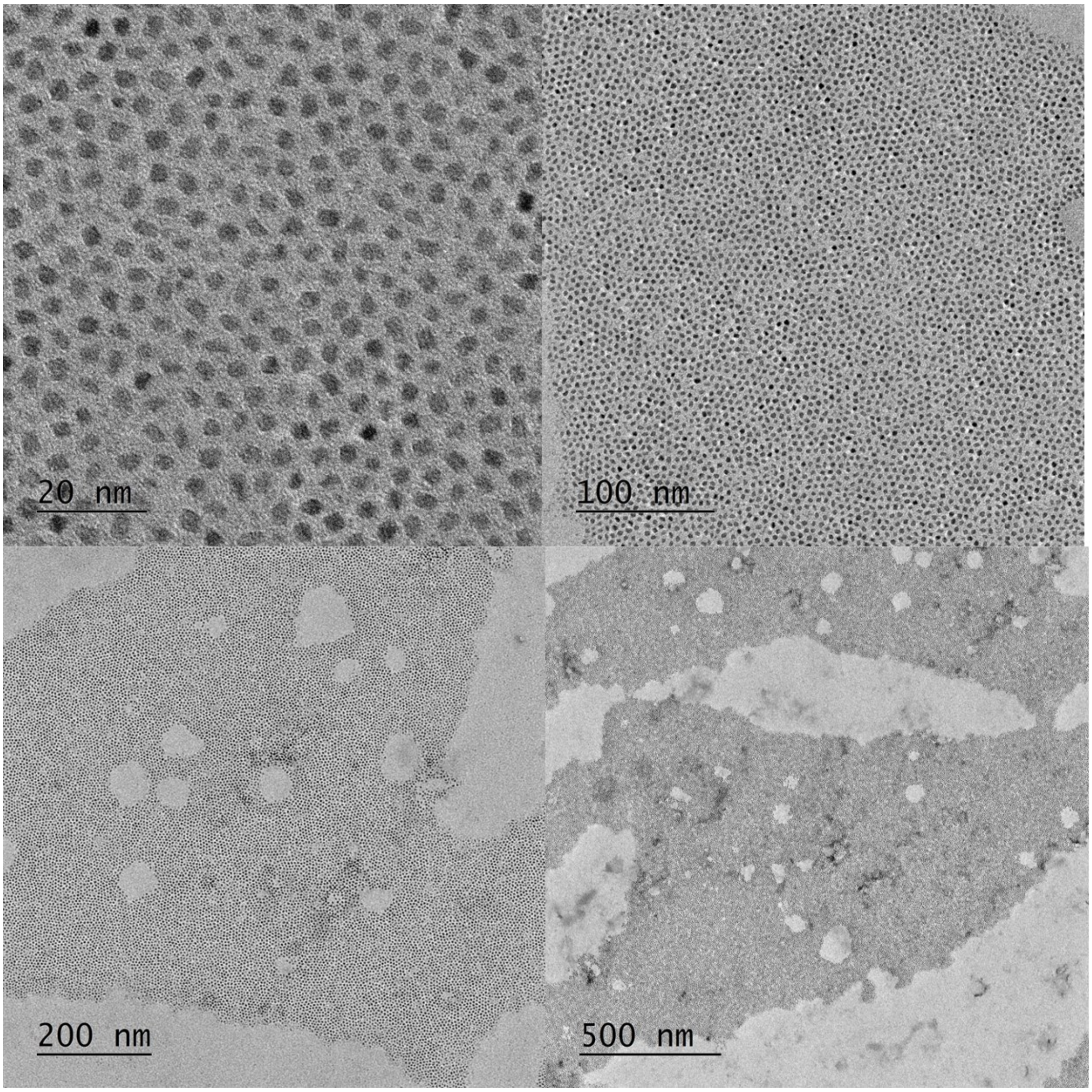

Figure S2: HfO_2 nanoparticles from HI-TOPO that show high monodispersity even over large areas.

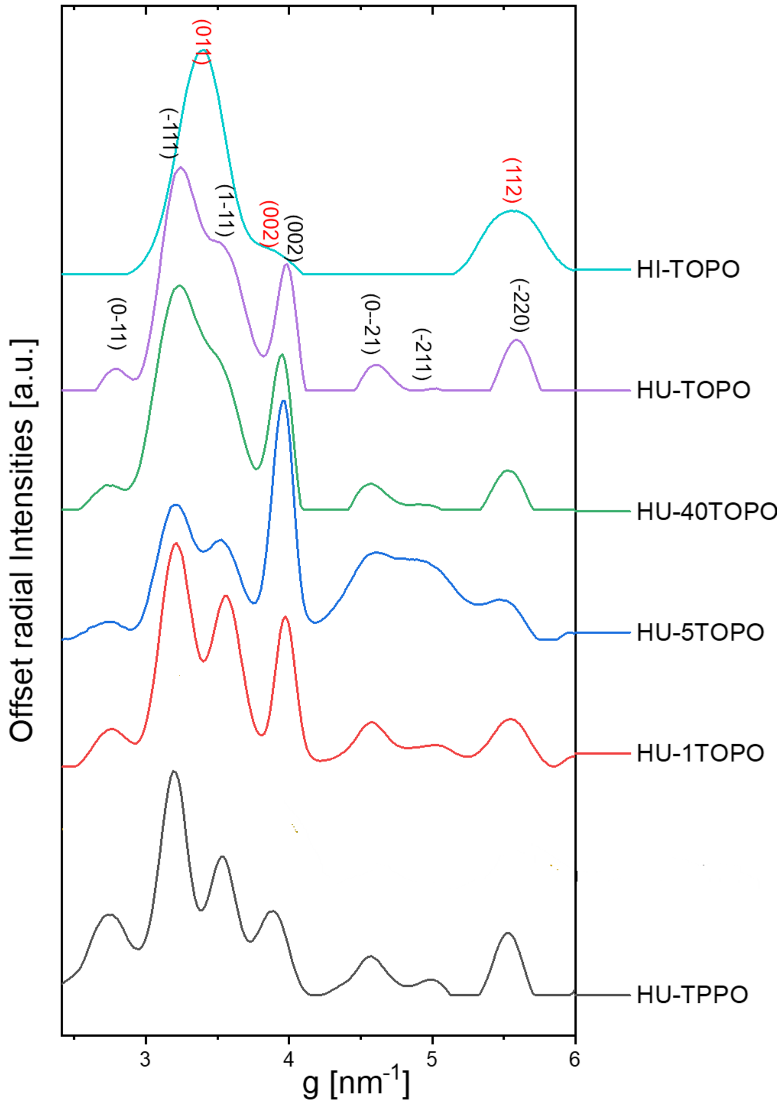

Figure S3: Selected area electron diffraction (SAED) analysis of the different synthesis approaches. HI-TOPO out of all samples resembles the tetragonal phase (the fluorite structure cannot be ruled out due to its similarity). The samples HU-TOPO, HU-40TOPO and HU-5TOPO correspond to the HfO_2-NR morphology which is featured in the diffraction intensities, where the (002) reflection is more intense and sharper compared to the others. This is due to the (002)-dimensional expansion of the particles. This effect becomes less relevant for the broadened morphologies of the HfO_2-NP (i.e. HU-1TOPO and HU-TPPO).

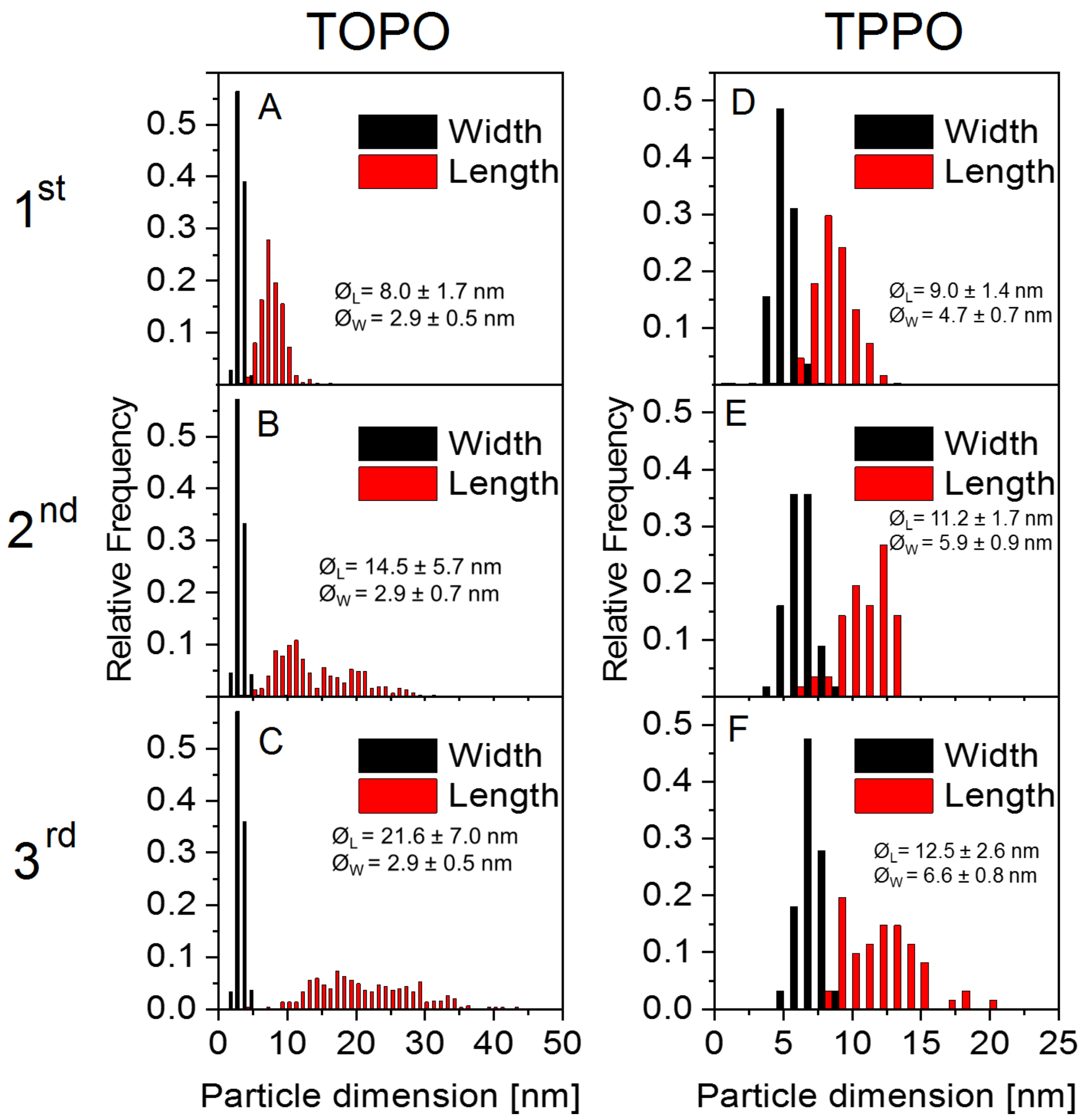

Figure S4: Width and length distribution of HfO_2-NR after one (A), two (B) and three (C) precursor additions within TOPO. Correspondingly, for D-E show the size distribution of HfO_2-NP within TPPO (for each, E and F, 65 particles were measured).

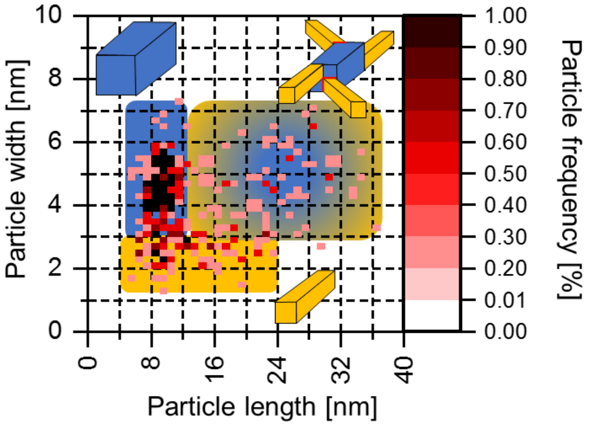

Figure S5: Size distribution frequency analysis of the HfO_2-NH sample. The colored areas indicate either the sizes of HfO_2-NP (blue), HfO_2-NR (orange) or HfO_2-NH (blue and orange mix).

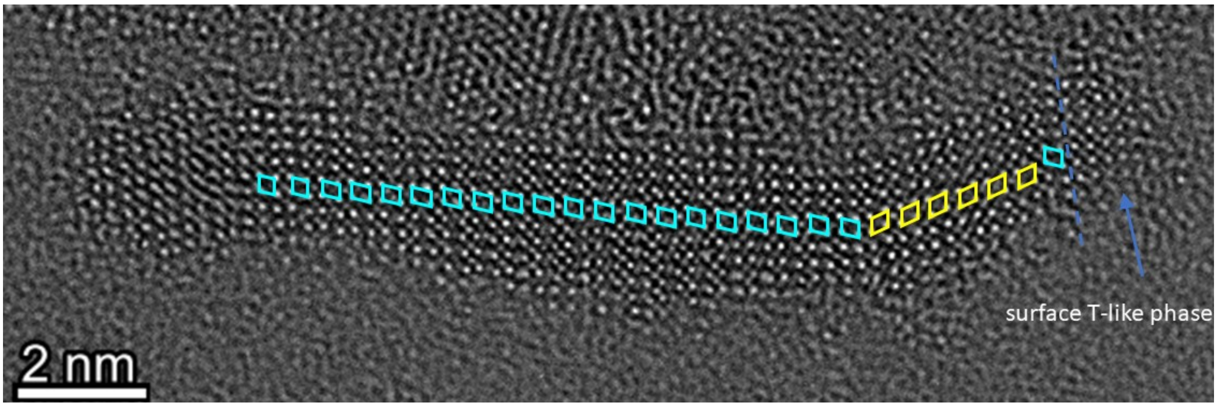

Figure S6: Example of a HfO_2-NR recorded from the <010> projection that shows monoclinic domains (cyan and yellow rhombs) and a tip end in a tetragonal-like phase (emphasized by dark blue ticked line).

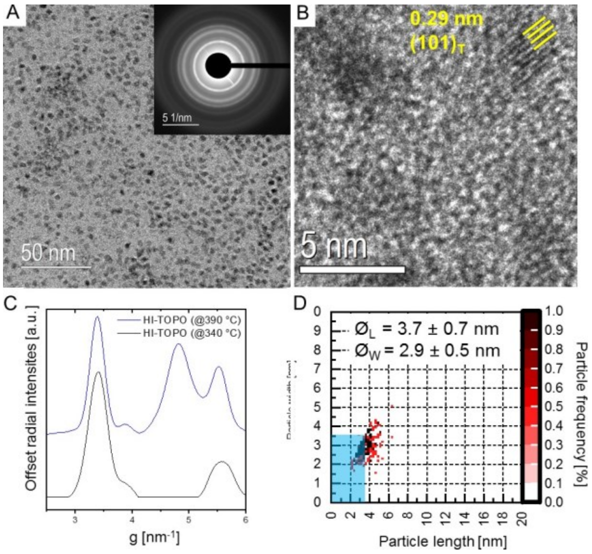

Figure S7: (A) Low magnification TEM image with selected area electron diffraction insert for HI-TOPO at 390 °C. (B) High resolution TEM image with (101) lattice spacing. (C) Comparison of HI-TOPO at 390 °C and HI-TOPO at 340 °C. The 390 °C experiment has an additional broad reflection at 4.7 nm^{-1} which can be attributed to the amorphous signal of the carbon grid. (D) Particle size distribution of HI-TOPO at 390 °C.

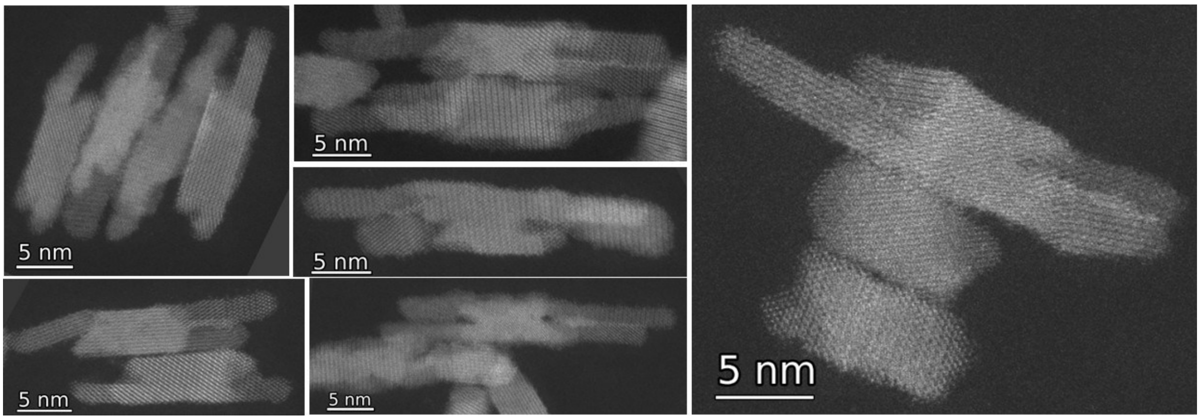

Figure S8: HAADF TEM images of HfO_2-NH, exposing rodlike extensions of nanoprisms.

Table S2: Surface energies (γ) of low index surfaces in monoclinic HfO_2

Facet	Calculated γ from literature [Jm^{-2}]
$(\bar{1}11)$	0.993,[3] 1.04[4]
(111)	1.199,[3] 1.25,[4] 1.25[5]
$(\bar{1}01)$	1.322,[3]
(110)	1.388,[3] 1.38[5]
(001)	1.416,[3] 1.45,[4] 1.51[5]
(011)	1.484,[3]
(101)	1.55,[3] 1.57[3]
(100)	1.667,[3] 1.79,[4] 1.67[5]
(010)	1.878,[3] 1.88[3]

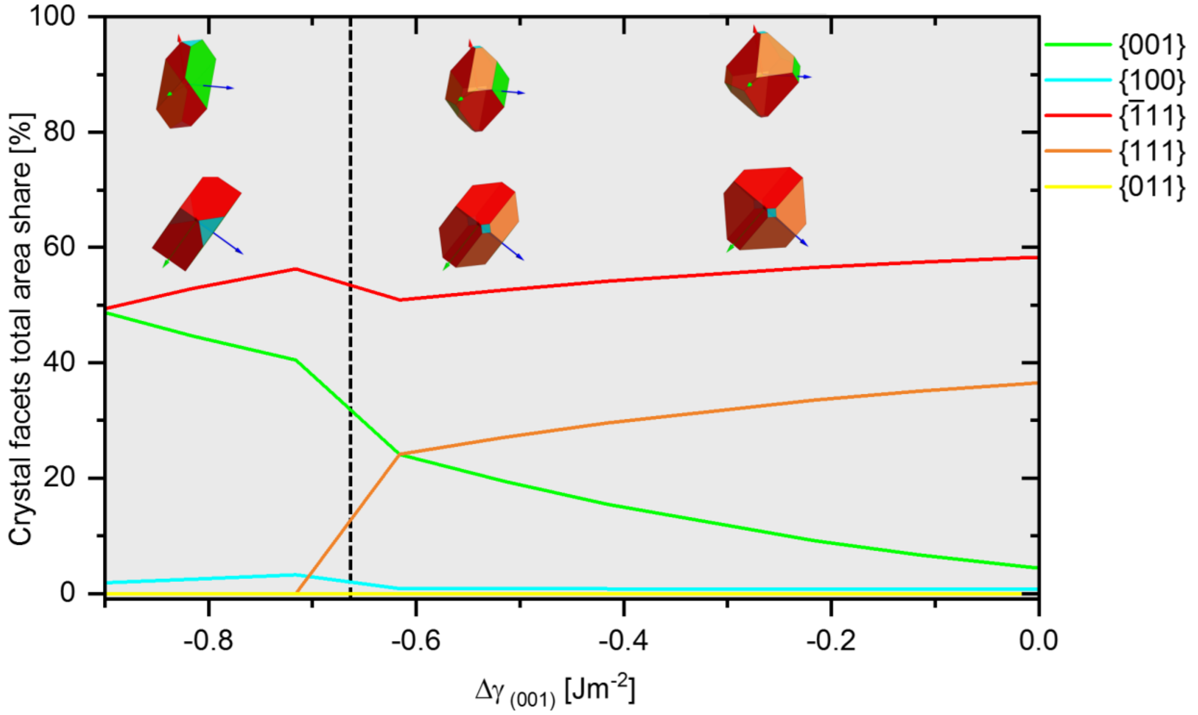

Figure S9: Simulated Wulff-construction area share of each facet which is plotted against the change of Δγ(001). Specific energy regions are separated with dashed lines with corresponding simulations of the crystal morphology (top one from a general crystal projection, bottom one from the <100> zone-axis). None of these morphologies have been observed in TEM. All Wulff-construction analyses have been performed with the WulffMaker Software with γ parameters based on the DFT calculations by Mukhopadhyay et al.. [3, 6]

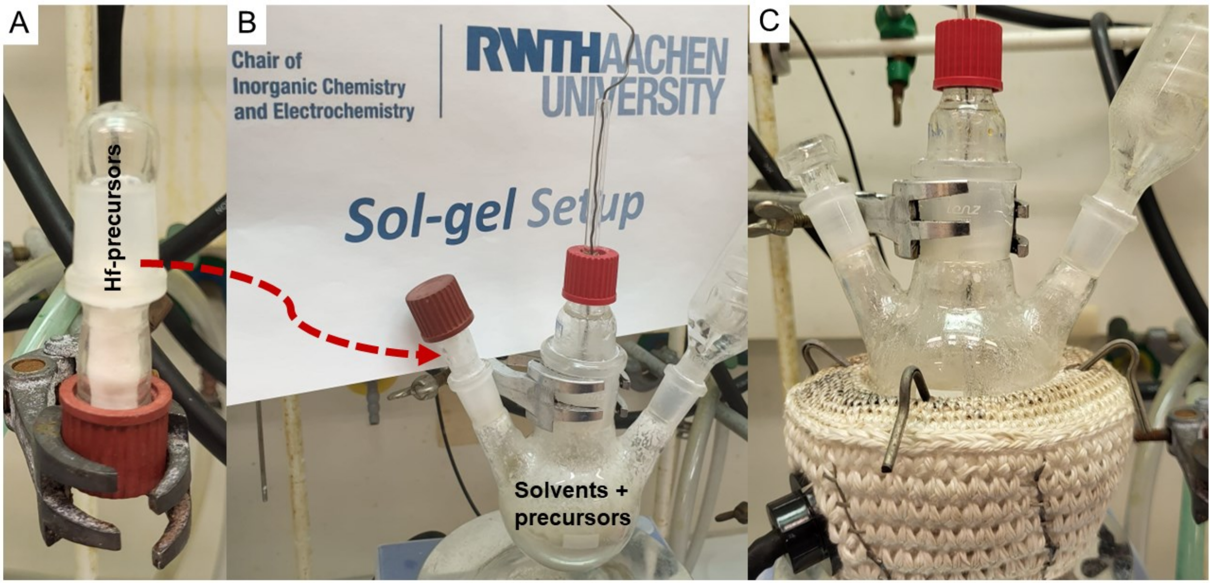

Figure S10: Transfer piece filled with solid precursor mixture stored under argon atmosphere (A). Three-necked round-bottom flask with connected transfer piece (B). Reaction set-up with heating mantle.

References

[1] J. E. Jaffe, R. A Bachorz, M. Gutowski, *Physical Review B* **2005,** *72*, 144107.

[2] K. R. Whittle, G. R. Lumpkin, S. E. Ashbrook, *Journal of Solid State Chemistry* **2006,** *179*, 2, 512.

[3] A. B. Mukhopadhyay, J. F. Sanz, C. B. Musgrave, *Physical Review B* **2006**, *73*, 11, 115330.

[4] X. Luo, A. Demkov, D. Triyoso, P. Fejes, R. Gregory, S. Zollner, *Physical Review B* **2008**, *78*, 24 245314.

[5] R. Batra, H. D. Tran, R. Ramprasad, *Applied Physics Letters* **2016**, *108*, 17, 172902.

[6] R. V. Zucker, D. Chatain, U. Dahmen, S. Hagège, W. C. Carter, *Journal of Materials Science* **2012**, *47*, 24 8290.
